# Exploring Social Biomarkers in High-Functioning Adults with Autism and Asperger’s Versus Healthy Controls: A Cross-Sectional Analysis

**DOI:** 10.1007/s10803-020-04493-5

**Published:** 2020-04-11

**Authors:** Marta Del Valle Rubido, Eric Hollander, James T. McCracken, Frederick Shic, Jana Noeldeke, Lauren Boak, Omar Khwaja, Shamil Sadikhov, Paulo Fontoura, Daniel Umbricht

**Affiliations:** 1 Roche Innovation Center Basel, Roche Pharmaceutical Research and Early Development, NRD, Basel, Switzerland; 2 Psychiatry and Behavioral Sciences, Albert Einstein College of Medicine and Montefiore Medicine, Bronx, NY USA; 3 Psychiatry and Behavioral Sciences, David Geffen School of Medicine at UCLA, Los Angeles, CA USA; 4 Center for Child Health, Behavior and Development, Seattle Children’s Research Institute, Seattle, WA USA; 5 Department of Pediatrics, University of Washington, Seattle, WA USA; 6 Roche Product Development Neuroscience, Basel, Switzerland; 7 Roche Global Product Strategy Neuroscience, Basel, Switzerland

**Keywords:** Biomarker, Eye movement, Olfactory, Social cognition

## Abstract

Biomarkers for autism spectrum disorder (ASD) are lacking but would facilitate drug development for the core deficits of the disorder. We evaluated markers proposed for characterization of differences in social communication and interaction in adults with ASD versus healthy controls (HC) for utility as biomarkers. Data pooled from an observational study and baseline data from a placebo-controlled study were analyzed. Between-group differences were observed in eye-tracking tasks for activity monitoring, biomotion, human activity preference, composite score (*p* = 0.0001–0.037) and pupillometry (various tasks, *p* = 0.017–0.05). Impaired olfaction was more common in the ASD sample versus HC (*p* = 0.018). Our preliminary results suggest the potential use for stratification and response sub-analyses outcome-prediction of specific eye-tracking tasks, pupillometry and olfaction tests in ASD trials

**Electronic supplementary material:**

The online version of this article (10.1007/s10803-020-04493-5) contains supplementary material, which is available to authorized users.

Autism spectrum disorder (ASD) is a highly heterogeneous neurodevelopmental disorder affecting at least 1 in 59 children in the US (Baio et al. 2018), or one in 132 people worldwide (Baxter et al. 2015). Individuals with ASD exhibit impaired social interaction and communication, restricted interests, repetitive behaviors, and unusual sensory responses (American Psychiatric Association 2013). Current treatment options rely heavily on behavioral interventions (e.g., applied behavior analysis) that aim to foster learning and skill development as well as to manage maladaptive behaviors, while medications such as atypical antipsychotics, psychostimulants, guanfacine and other mediations that are unapproved for ASD such as selective serotonin reuptake inhibitors and anti-convulsants, are frequently employed to reduce associated behaviors of irritability/aggression and inattentive/hyperactive behaviors (Zwaigenbaum et al. 2015). However, core ASD deficits tend to persist despite intervention, and no pharmacotherapies possess proven efficacy at reducing the core symptoms of deficits in social interaction and communication, and restricted and repetitive behaviors (Ji and Findling 2015).

Drug development for the core social deficits of ASD faces various challenges: first, as for many neuropsychiatric disorders, a lack of surrogate markers (i.e., biomarkers) able to detect therapeutic efficacy is a key obstacle (Anagnostou et al. 2015; Baxter et al. 2015; Brugha et al. 2015; Zwaigenbaum et al. 2015, 2013). Common approaches available to quantify social communication deficits in individuals with ASD were not developed with the intent for use in ASD and are cumbersome and subject to bias, as they are based on caregiver report (Anagnostou et al. 2015). A second challenge unique to neurodevelopmental disorders like ASD concerns the fact that the initial evaluation of novel compounds usually takes place in clinical trials in adults, rather than in trials in the ultimate optimal target population of children and adolescents. However, deficits that are commonly described in children and adolescents with ASD in social cognition, for example, skills of empathy, imagination, theory of mind (TOM; beliefs, desires, intentions, and perspectives), social pragmatics and advanced language skills (Williams White et al. 2007) are known to show changes across different stages of development in longitudinal studies (Sarrett and Rommelfanger 2015). Thus, the profiles of abnormalities and corresponding surrogate markers for therapeutic efficacy may be different across the life-span. In addition, clinical heterogeneity of ASD is presented in a variety of symptom profiles, severity (Lai et al. 2013) and levels of intellectual and functional communication ability and constitutes a major obstacle both to the diagnosis and treatment of ASD (Charman et al. 2017; Jeste and Geschwind 2014; Masi et al. 2017). Furthermore, diagnostic scales used in ASD target relatively heterogeneous groups of behaviors and were not originally developed to sensitively assess social communication or more narrow components of social responsiveness in the context of a clinical trial. To date, results from contemporary investigations attempting to characterize and group ASD social and communication impairments and link them mechanistically to biologically proximal information-processing functions have been mixed; no single biomarker or cognitive domain has emerged as a primary thus far. Therefore, it is paramount to identify stratification factors that are easily assessed in a clinical setting and that reduce the autistic symptom variance. Overall, few studies have attempted to assess the discriminant properties, reliability and validity of putative markers of core deficits as treatment biomarkers with utility for clinical trial application.

The current work aims to assess the discriminant validity of several promising surrogate markers or social functioning in high-functioning adults with ASD and in healthy volunteers (i.e. observe if the direction of difference is as expected). The measures in the study were selected based on their ability to objectively evaluate different system levels of social cognition and communication in a multi-dimensional approach with the expectation that a fragmentation of social communication processes in ASD would allow for the identification of the measures that best relate to neurobiological or neurocognitive processes and to the disease and/or symptom severity. These measures included the eye‐tracking paradigms and olfaction, representing a basic level of screening, attunement to, and extraction of, socially relevant information and the Affective Speech Recognition test (ASR) and Reading‐the‐Mind‐in‐the‐Eyes Test (RMET) as an intermediate level corresponding to the ability to capture and process composite information critical for social communication. The results of this work will help to interpret data from multicenter clinical trials and to build a well-characterized battery of objective assessments from which to choose from for future clinical trials contingent on the mechanism of action and the expected pharmacodynamic effect of a drug.

As an exploratory objective, a *post-hoc* analysis evaluated the utility of one of these surrogates, the Sniffin’ Sticks Screening 12 olfaction identification test (Kobal et al. 1996), as a stratification factor and a predictor of deficits in social interaction and communication. The Sniffin’ Sticks Screening 12 olfaction identification test was chosen because olfaction plays an important role in social communication in humans (Hays 2003; Stevenson 2010; Wysocki and Preti 2004) and compared to other exploratory measures, it is the only one for which normative data to classify subgroups exists (Kobal et al. 1996). This test was also selected in the context of the development of the vasopressin antagonist RG7713 in the phase 1 clinical study NCT01474278 (Umbricht et al. 2016) given the evidence of high expression of V1a receptor (V1aR) in the ventral and lateral portion of the anterior olfactory nucleus, different structures of the olfactory bulb and an olfactory (piriform) cortex and presence of V1AR mRNA in endothelial cells of midline blood vessels between the main olfactory bulbs in rats (Ostrowski et al. 1994). In ASD, altered behavioral responses to social chemosignals have been reported (Endevelt-Shapira et al. 2018) implicating olfaction as a potential factor guiding neurodevelopment (Secundo et al. 2014). These reports also point towards a possible involvement for olfaction in abnormal processing of socially salient information and/or providing a biomarker indexing disruptions of the embryogenic development within critical time frames (Rozenkrantz et al. 2015). For these reasons, we assessed olfaction in high-functioning adults with ASD and healthy controls (HCs) and studied its relation to two fundamental aspects of social cognition: auditory and visual emotion recognition.

A companion manuscript describing the assessment of the concurrent validity of these exploratory assessments and the feasibility of implementation in a clinical study has recently been published (Del Valle Rubido et al. 2018) and helps to contextualize the findings of the present work. These exploratory measures showed varying associations across ASD severity, adaptive skills, and behavior, suggesting that each of the exploratory measures examined have the sensitivity to capture information that individually informs aspects of social functioning, but they appear to largely tap into functional differences that are at least partially independent.

## Methods

### Design

Two studies form the basis of this analysis: Study 1 (NCT01669889), a multicenter, observational study that enrolled 19 high-functioning adults with ASD or Asperger’s syndrome according to the Diagnostic and Statistical Manual-Fourth Edition (DSM-IVTR) (American Psychiatric Association 2000) and 19 HC; and Study 2, an intervention trial (NCT01474278) that enrolled 19 high-functioning participants with ASD or Asperger’s syndrome (Umbricht et al. 2016).

**Fig. 1:**
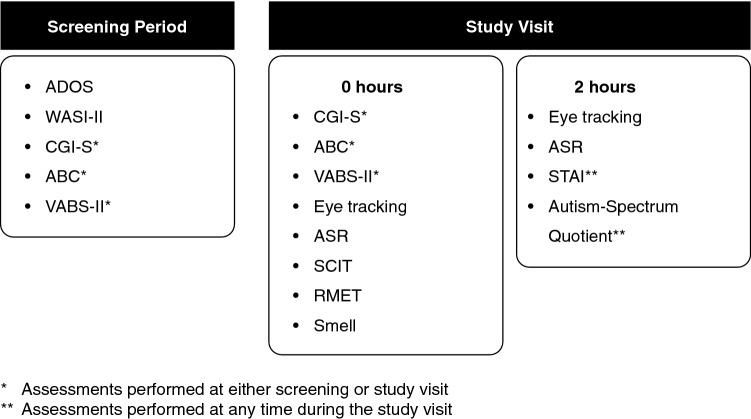
Study design and schedule of assessments. Identical protocols were performed in Studies 1 and 2, assessments are listed in the order performed. Order and number of test administrations were determined by the pharmacokinetic characteristics of the investigational drug and the burden to participants in Study 2. Some participants opted to combine screening and day 1 visits into a single visit. Assessments of clinical symptomatology (i.e. ABC, ADOS, CGI-S, SCIT and VABS-II) were only assessed in the ASD population at the screening visit. Social Communication Interaction Test (SCIT) results are not addressed in this manuscript. *ABC* aberrant behavior checklist, *ADOS* autism diagnostic observation schedule, *ASR* affective speech recognition, *CGI-S* clinical global impression, *RMET* reading the mind in the eyes, *SCIT* social communication interaction test, *STAI* state/trait anxiety inventory, *WASI-II* Wechsler Abbreviated Scale of intelligence II

Study 1 consisted of a screening period (maximum 35 days, completing diagnostic, clinical, and functional measures) followed by the Day 1 study visit (alternatively, participants were offered to combine the screening and study visits in a single day, if eligible). During the Day 1 visit, participants were seen in the clinical research unit and were discharged following completion of all assessments (Fig. 1). HC found to be free of current psychopathology, completed assessments according to an identical schedule as participants with ASD, except for the measurements of clinical symptomatology at screening. Study 2 obtained data from baseline assessments prior to the administration of intravenous drug or placebo, which were combined with data from the participants with ASD or Asperger’s syndrome who participated in Study 1. No participants participated in both protocols. The same assessments were performed the same number of times and in the same order and schedule in both studies (Fig. 1).

### Inclusion Criteria

High-functioning (intelligence quotient [IQ] > 70) male patients (18–45 years old) with a diagnosis of ASD or Asperger’s syndrome (DSM-IVTR) (American Psychiatric Association 2000) by clinical evaluation by an experienced psychologist or psychiatrist and confirmed by scores obtained from the administration of the Autism Diagnostic Observation Schedule (ADOS) (Lord et al. 2002) by a trained clinician. Age-matched healthy males were enrolled in both studies. Full inclusion/exclusion criteria are presented in ESM Table 1 (online resource 1).

**Table 1:** Baseline characteristics of participants in Studies 1 and 2

Variable	ASD Mean (SD) N = 38	HCs Mean (SD) N = 19	Result interpretation	
			Scoring ranges	
Age in years	24.2 (5.8)	26.7 (4.3)	18–40	
WASI-II				
FSIQ	101.1 (14.3)	117.7 (9.7)	Normal range: Mean = 100, SD = 15	Higher scores, better skills/milder symptoms
VIQ	100.5 (16.1)	117.6 (12.0)		
PIQ	100.6 (12.7)	113.5 (11.0)		
VABS-II				
Adaptive behavior composite	63.8 (11.6)	–	Normal range: Mean = 100, SD = 15	
Communication	64.4 (18.1)	–		
Daily living skills	68.7 (11.9)	–		
Socialization	64.6 (13.1)	–		
ADOS Module 4				
Total	12 (3.8)	–	0–32	Higher scores, worse skills/more severe symptoms
Communication	3.0 (1.3)	–	0–8 (ASD cut-off = 2)	
Social interaction	6.7 (2.1)	–	0–14 (ASD cut-off = 4)	
Communication and social interaction	9.6 (3.0)	–	0–22 (ASD cut-off = 7)	
ABC				
Total	32.9 (20.9)	–	0–174	
Irritability	5.9 (6.5)	–	0–45	
Lethargy/social withdrawal	11.5 (7.4)	–	0–48	
Stereotypic behavior	3.6 (3.6)	–	0–21	
Hyperactivity	9.0 (8.1)	–	0–48	
Inappropriate speech	2.9 (2.7)	–	0–12	
STAI	38.7 (13.5)	31.0 (9.1)	20–80	
CGI-S	4.1 (0.6) 4 = moderately ill	–	1 = normal, not at all ill; 7 = amongst the most extremely ill	
AQ	28.53 (7.2)	14.21 (5.8)	0–50	

All participants were male

*ABC* Aberrant behavior checklist, *ADOS* autism diagnostic observation schedule, *AQ* autism quotient, *ASD* autism spectrum disorder, *CGI* clinical global impression-severity, *FSIQ* full-scale intelligence quotient, *HC* healthy control, *PIQ* performance intelligence quotient, *SD* standard deviation, *STAI* state/trait anxiety inventory, *VABS-II* Vineland Adaptive Behavior Scale-II, *VIQ* verbal intelligence quotient, *WASI-II* Wechsler Abbreviated Scale of Intelligence version II

### Clinical and Functional Measures Administered

#### Wechsler Abbreviated Scale of Intelligence Version II (WASI-II) (Wechsler 2008)

A brief and reliable measure of adult intelligence, yielding estimates of verbal intelligence quotient (VIQ), performance intelligence quotient (PIQ) and full-scale intelligence quotient (FSIQ).

#### ADOS Module 4 (Lord et al. 2002) (ASD Group Only)

A validated, examiner-rated, structured instrument that systematically prompts assessment of social behavior and interaction for evaluation of ASD diagnoses. Scoring yields three domains: communication, social interaction, and the combined communication and social interaction score. Standard published scoring cutoff scores were applied; higher scores indicate greater levels of core deficits.

#### Aberrant Behavior Checklist-Community Version (ABC-C) (Aman et al. 1985)

An informant-rated, 58-item questionnaire normed for developmentally disabled populations. Ratings generate scores on five factors: irritability, lethargy and social withdrawal, stereotypic behavior, hyperactivity/non-compliance and inappropriate speech. Items are scored from 0 (no problem) to 3 (severe problem).

#### Vineland Adaptive Behavior Scale-II (VABS-II) (Sparrow 2011) (ASD Group Only)

A caregiver-rated, semi-structured interview to assess adaptive behavior and skills in developmental disorders. Measures adaptive behavior across subscales of communication, daily living skills, and socialization. A composite score is generated as a measure of overall functioning. Higher scores correspond to better adaptive behavior skills.

#### Clinical Global Impression-Severity (CGI-S) (Guy 1976) (ASD Group Only)

A clinician-rated measure of overall illness severity. Ratings are made on a seven-point scale ranging from one (normal) to seven (among the most extremely ill patients).

#### Autism Spectrum Quotient (AQ) (Baron-Cohen et al. 2001a, b) (Study 1 Only)

A self-report measure for normally functioning individuals, assessing autistic traits. It includes 50 items that assess communication, social skills, imagination, attention to detail and attention switching. Higher AQ scores indicate more autistic traits and cognitive characteristics; in surveys, AQ scores greater than 32 are highly discriminating between individuals with ASD compared with non-affected individuals.

#### State/Trait Anxiety Inventory-State (STAI) (Spielberger 2010)

A measure of state (i.e. situational) and trait (stable, constitutional) anxiety, consisting of 20 self-completed items scored on a Likert scale of one (not at all) to four (very much so). Higher STAI scores indicate greater anxiety.

### Exploratory Assessments

#### Eye Tracking

Eye tracking has been shown to be a robust technique for studying processes associated with social impairments in individuals with ASD and for quantifying differences in looking behaviors between individuals with ASD and HCs (Chita-Tegmark 2016; Frazier et al. 2017). A Tobii T60XL 60-Hz eye-tracking system was used to measure gaze positions of participants during the following paradigms:

(1) Activity monitoring (Shic et al. 2011; Frederick Shic et al. 2014): Participants viewed videos of two actresses performing simple social activities involving joint play with visually salient distracters in the background. Dependent variables included the ratio of time spent looking at heads, bodies, activities, and backgrounds relative to total time spent looking at the scenes.

(2) Biological motion preference task (biomotion) based on Annaz et al. 2012 (Annaz et al. 2012): Participants watched multiple trials of various side-by-side videos containing, in pseudorandomized left–right order, dynamic point-light displays of a human actor’s performance of an activity (e.g., walking, jumping a rope, waving: biomotion condition, i.e., biological motion) (Carnegie Mellon University Graphics Lab) alongside computer-generated animations of moving dots (control condition). Dependent variables included ratio of looking to biomotion over looking at either biomotion or the control condition; and proportion of times participants oriented to biomotion first compared with control first.

(3) Biological motion detection (biodetection) (Kaiser et al. 2010): Under masked and unmasked conditions, participants were shown videos of biological or mechanical movement. Participants were asked to identify whether a human being was walking or not. The dependent variable was *d*-prime, a measurement of signal detection ability.

(4) Complex dynamic social task (Klin et al. 2002): Participants were shown a series of short clips from the movie Who’s Afraid of Virginia Woolf (WAVW), which displayed scenes with complex social interactions with high emotional tone. Dependent variables included attention (% of total looking time) to: eyes of the actors; mouths of the actors; the bodies of the actors; and background regions including objects.

(5) Gaze and (6) Gender discrimination in a static face scanning task (Andari et al. 2010): Participants viewed a series of static photographs of human faces with instructions to either identify gaze direction (direct/averted) or gender (male/female). Primary dependent variables included attention (percentage of total looking time) to facial region and non-facial regions. Secondary dependent variables included time spent looking at the mouth and eyes of the presented faces.

(7) Human activity preference task (Pierce et al. 2011): In this preferential looking task, two videos were presented simultaneously, side-by-side, on a single screen, with one video depicting a human performing an activity (Human) and the other video a computer-generated, continuously moving/changing geometric pattern (Geometry). The left–right ordering of videos was counter-balanced. The dependent variable was attention (percentage of looking) to Human compared with Geometry. This paradigm is referred to as the GeoPref task by the paradigm designers (Pierce et al. 2015), however, for simplicity and consistency with the outcome measure we refer to this paradigm as the Human Activity preference task.

A composite score was defined a priori by one of the current authors (FS), derived from the key parameters of all tasks, with weights based loosely upon results collected in a separate pilot study conducted prior to the current study. This composite score was intended to capture orientation and attention to socially relevant information, with lesser emphasis on the human activity preference and WAVW tasks and least emphasis on biological motion detection ability. Trials were considered valid if they contained more than 70% valid gaze looking time collected during the stimulus presentation, and/or exhibited a calibration uncertainty of less than 1.5 degrees in the trial, or less than 3 degrees over the entire session (Shic 2008). A participant’s session data were considered valid for a paradigm if it contained 50% or more valid trials.

#### Pupillometry (Anderson et al. 2013)

Change in pupil diameter during the performance of eye-tracking paradigms was integrated into the apparatus. Differences in tonic pupil size indicate level of arousal in response to stimulus presented (Laeng et al. 2012) and children with ASD have been shown to have larger tonic pupil sizes than HCs (Anderson and Colombo 2009). In our study, we did not consider dynamic changes, only tonic pupil sizes.

#### Reading the Mind in the Eyes Test-Revised (RMET) (Baron-Cohen et al. 1997; Baron-Cohen et al. 2001a, b)

A measure of TOM and facial affect processing, RMET scores have been found to discriminate patients with ASD from typical controls (Baron-Cohen et al. 2001a, b). Participants are presented with 36 different pictures of the eye region of human faces. Participants then have to select one of four different emotion labels that describe the emotion the person is feeling (Baron-Cohen et al. 2001a, b). Higher scores indicate greater TOM and detection of emotion.

#### Affective Speech Recognition (ASR) (Hollander et al. 2007)

A measure of emotion recognition ability, determined by accuracy of identification of affective speech. Participants are played a recording of four sentences of neutral content with eight different emotional intonations (angry, disgusted, fearful, happy, lustful, neutral, sad and surprised). Each intonation is repeated six times for a total of 48 sentences. Participants have to select the appropriate emotion from a list (Hollander et al. 2007). Higher scores indicate better emotion recognition. ASR performance has been described as impaired in patients with right temporoparietal lesions (Heilman et al. 1975), and ASR scores showed possible improvements after intravenous oxytocin compared with placebo administration in adults with ASD (Hollander et al. 2007).

#### Olfactory Measure Sniffin’ Sticks Screening 12 test (Hummel et al. 2007; Kobal et al. 1996)

Participants are asked to smell twelve Sniffin’ Sticks (pen-like devices containing different odors), one at a time, and to select the correct odor descriptor from a selection of four possible choices. One point is scored for each correct answer (maximum score 12). Participants were instructed not to eat, to drink only water and to avoid chewing gum or smoking cigarettes at least fifteen minutes before the test. A brief history was collected regarding the participant’s current allergies and nasal congestion in order to ensure validity in the test results. All participants were considered evaluable by the investigators at the time of testing. Based on their scores, participants were grouped into normal (normosmic) and impaired (< 10 points) according to normative data, and further subgrouped into hyposmic (< 10– > 4 points) and anosmic (< 4 points) (Kobal et al. 1996).

### Statistical Analysis

All statistical analyses were conducted with SAS software (SAS Institute Inc., Carey, NC USA) and R (R Foundation for Statistical Computing, Vienna, Austria). An analysis of covariance model was used to estimate mean differences between ASD and HC adjusting for age and FSIQ. The FSIQ, a comprehensive and broadly used measure of general cognitive and intellectual functioning also frequently used as part of the diagnosis of intellectual disabilities, was selected as covariate. These differences and their standard errors were estimated from the models. The ANCOVA model used in the analyses assumes equal variances between the two groups. For eye tracking and pupillometry measures, an additional random subject effect was introduced into the model in order to account for correlations between the two-repeated measurements. Cohen’s f-squared was used as a measure of the effect size for group differences. For comparisons of normosmic vs. hyposmic ASD subjects an ANOVA model with a group as a fixed effect was used. To maintain an experiment-wise error rate of alpha of 0.05 when doing multiple comparisons, we used the Bonferroni correction. The adjusted threshold for significance is alpha of 0.00094*.* Due to the exploratory nature of the analyses, *p*-values should be interpreted as descriptive measures of trend, rather than determinants of statistical significance and with caution.

### Ethics

Both studies were conducted in accordance with the principles of the Declaration of Helsinki and Good Clinical Practice. The study sites were the Albert Einstein College of Medicine, Bronx, NY, USA; the UCLA Semel Institute CAN Clinic, Los Angeles, CA, USA; and the Child Study Center at Yale University School of Medicine in New Haven, CT, USA. Study protocols were reviewed and approved by the institutional review boards of each institution.

## Results

Baseline characteristics for all participants are shown in Table 1. Recruitment was intended to include IQ-matched HCs, however, although mean age was similar, mean IQ was higher in the HC group (Table 1). Median FSIQ score was 116.0 in the HC group (range 101.0–140.0) and 100.0 in the ASD group (range 71.0–136.0).

### Between-Group Comparisons

#### Baseline Assessments of Clinical Symptomatology: AQ and STAI

From the assessments performed in both ASD and HC groups, the AQ was the assessment which showed the greatest effect size between ASD and HC (ASD group [least squares mean] 29.72; HC group: 13.01; Δ = 16.71; f^2^ = 1.3; *p* < 0.001; t = 6.66; df = 34). However, no differences were observed in the overall level of anxiety measured by the STAI total score (ASD group 38.69; HC group: 31.09; Δ = 7.60; f^2^ = 0.062; *p* = 0.076; t = 1.81; df = 53).

### Exploratory Assessments

#### Eye Tracking

Based on the criteria for valid trials, 86.2% of trials from the ASD group and 85.0% of trials from the HC group were valid. No between-group differences in trial acquisition were present in any eye-tracking outcome measure.

**Fig. 2:**
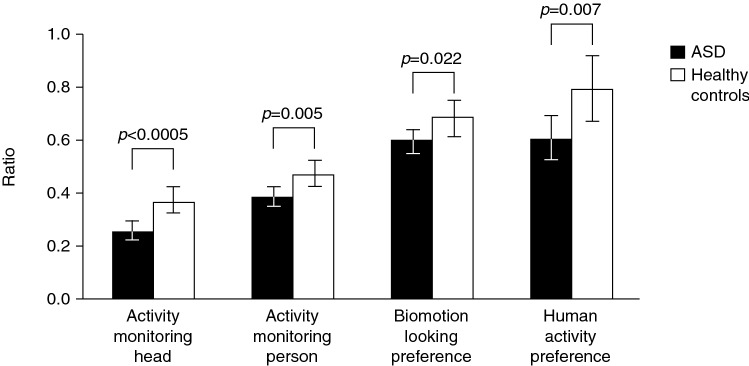
Eye tracking results for participants with ASD and HCs. *P*-values less than 0.00094 are considered statistically significant after multiplicity adjustment. Data are estimated mean ratios ± 90% confidence intervals for the amount of time spent looking at a specific area of interest and the total amount of time looking at the whole screen for each paradigm. *ASD* autistic spectrum disorder, *HC* healthy control

Results showed differences (ASD-HC) in the expected direction for activity monitoring, biomotion, human activity preference (social compared with geometry) and composite score (Fig. 2, Table 2). In activity monitoring, participants with ASD spent less time looking at the head (Δ = –0.11; [90% CI − 0.16 to − 0.07]; f^2^ = 0.65; *p* < 0.0005; t = 4.04; df = 48); and the person (Δ = –0.09; [90% CI − 0.14 to − 0.04]; f^2^ = 0.52; *p* = 0.005; t = 2.94; df = 48) than HC (Table 2). In the biomotion task, participants with ASD showed less preference for biological motion compared with control stimuli (Δ = –0.09; [90% CI − 0.15 to − 0.03]; f^2^ = 0.35; *p* = 0.02; t = − 2 = − 2.11; df = 50) (Table 2). In the human activity preference tasks, participants with ASD showed less preference for human activities as compared with geometric shape videos (Δ = − 0.19; [90% CI − 0.30 to − 0.08]; f^2^ = 0.32, *p* < 0.01; t = 2.8; df = 48). Finally, the composite score capturing key parameters across eye-tracking tasks was lower for participants with ASD compared with HC, indicating a general deficit affecting attention to socially relevant information (Δ = − 0.48; [90% CI − 0.86 to − 0.11]; f^2^ = 0.3, *p* = 0.04; t = 2.14; df = 49 = 49).

**Table 2:** Eye tracking data for ASD and HC groups

Task^a^	Mean ASD (N = 38)	Mean control (N = 19)	Estimate of difference ASD-HC	90% CI of estimate	*P* value	Cohen’s f^2^
*Activity monitoring*						
Activity (ratio)	0.36	0.31	0.05	− 0.01, 0.11	0.19	0.34
Background (ratio)	0.26	0.22	0.04	− 0.010, 0.08	0.20	0.13
Body (ratio)	0.13	0.11	0.03	− 0.0003, 0.05	0.11	0.16
Distractors (ratio)	0.13	0.10	0.03	− 0.006, 0.06	0.17	0.16
Head (ratio)	0.25	0.37	− 0.11	− 0.16, − 0.07	0.00019^b^	0.65
Person (ratio)	0.38	0.47	− 0.09	− 0.14, − 0.04	0.005	0.52
*Biodetection*						
*d*-prime (masked condition)	1.53	1.58	− 0.06	− 0.65, 0.54	0.88	0.21
Latency (ms)	300.37	274.34	26.02	− 20.23, 72.28	0.36	0.11
Looking preference (ratio)	0.60	0.69	− 0.09	− 0.15, − 0.03	0.02	0.35
Orienting preference (ratio)	0.49	0.50	− 0.01	− 0.05, 0.03	0.62	0.03
*WAVW*						
Background (ratio)	0.13	0.12	0.01	− 0.01, 0.04	0.39	0.20
Body (ratio)	0.10	0.08	0.02	− 0.01, 0.05	0.28	0.21
Eyes (ratio)	0.40	0.42	− 0.03	− 0.11, 0.06	0.59	0.10
Head (ratio)	0.74	0.78	− 0.03	− 0.09, 0.02	0.27	0.22
Mouth (ratio)	0.25	0.26	− 0.01	− 0.09, 0.06	0.76	0.09
*Gaze discrimination*						
Eyes (ratio)	0.38	0.37	0.01	− 0.07, 0.09	0.84	0.04
Inside face (ratio)	0.92	0.94	− 0.02	− 0.07, 0.02	0.38	0.049
Mouth (ratio)	0.06	0.06	0.008	− 0.03, 0.05	0.71	0.01
Nose (ratio)	0.33	0.38	− 0.05	− 0.12, 0.01	0.19	0.13
*Gender discrimination*						
Eyes (ratio)	0.22	0.18	0.04	− 0.02, 0.09	0.33	0.14
Inside face (ratio)	0.91	0.93	− 0.03	− 0.08, 0.03	0.39	0.05
Mouth (ratio)	0.09	0.07	0.02	− 0.02, 0.07	0.42	0.06
Nose (ratio)	0.45	0.54	− 0.09	− 0.16, − 0.01	0.06	0.20
*Human activity preference (Social vs Geometric)*						
Human activity preference (ratio)	0.60	0.79	− 0.19	− 0.30, − 0.08	0.007	0.32
*Total*						
Composite score	− 0.08	0.41	− 0.48	− 0.86, − 0.11	0.04	0.30

Estimate refers to estimated mean differences between ASD and HC derived from an analysis of Covariance model

P values less than 0.00094 are considered statistically significant after multiplicity adjustment

^a^Categories in each task (e.g. Activity, Background) refers to the time spent looking at this aspect of the visual scene in relation to the overall looking time

^b^Measure that survived Bonferroni correction

*ASD* Autism spectrum disorder, *HC* healthy control, *WAVW* Who’s afraid of Virginia Woolf

#### Pupillometry

The ASD group had larger pupil sizes than the HC group during all seven eye tracking paradigms: activity monitoring (Δ = 0.35 mm; [90% CI 0.09 to 0.60]; f^2^ = 0.16; *p* = 0.03; t = 2.25; df = 50), d-prime (Δ = 0.33 mm; [90% CI 0.02 to 0.64]; f^2^ = 0.13, *p* = 0.09; t = 1.74; df = 49), biomotion preference (Δ = 0.33 mm; [90% CI 0.06 to 0.59]; f^2^ = 0.2; *p* = 0.05; t = 2.01; df = 49), gaze discrimination (Δ = 0.36 mm; [90% CI 0.10 to 0.63]; f^2^ = 0.17; *p* = 0.03; t = 2.25; df = 50), gender discrimination (Δ = 0.39 mm; [90% CI 0.13 to 0.64]; f^2^ = 0.2; *p* = 0.02; t = 2.45; df = 50), human activity preference (Δ = 0.29 mm; [90% CI 0.03 to 0.55]; f^2^ = 0.17; *p* = 0.07; t = 1.85; df = 49) and WAVW (Δ = 0.39 mm; [90% CI 0.11 to 0.67]; f^2^ = 0.22; *p* = 0.03; t = 2.26; df = 50) tasks (Table 3).

**Table 3:** Between-group comparisons of pupillometry data

Pupillometry	Mean ASD	Mean HC	Estimate of Difference ASD-HC	90% CI of estimate	*p* value	Cohen’s f^2^
Activity monitoring (mm)	3.65	3.30	0.35	0.09, 0.60	0.03	0.16
Biodetection (mm)	4.40	4.06	0.33	0.02, 0.64	0.09	0.13
Biomotion (mm)	4.22	3.89	0.33	0.06, 0.59	0.05	0.20
Gaze discrimination (mm)	3.59	3.23	0.36	0.10, 0.63	0.03	0.17
Gender discrimination (mm)	3.60	3.21	0.39	0.13, 0.64	0.02	0.20
Human activity preference (mm)	3.71	3.42	0.29	0.03, 0.55	0.07	0.17
WAVW (mm)	4.02	3.64	0.39	0.11, 0.67	0.03	0.22

Estimate refers to estimated mean differences between ASD and HC derived from an Analysis of Covariance model. P-values less than 0.00094 are considered statistically significant after multiplicity adjustment

*ASD* Autism spectrum disorder, *HC* healthy control, *WAVW* who’s afraid of virginia woolf

#### RMET and ASR

Participants with ASD showed similar performance on both the RMET and ASR total scores compared with HCs; RMET: ASD group 67.7%; HC group: 74.7% (Δ = − 6.98; [90% CI − 16.52 to 2.56]; f^*2*^ = 0.045*,; p* = 0.224; t = − 1.24; df = 34); ASR: ASD group 55.6% correct answers; HC group: 57.3% correct answers (Δ = − 1.67 [90% CI − 8.78 to 5.44]; f^2^ < *0.01; p* = 0.70; t = − 0.39; df = 52). In the ASR, the largest differences were observed for the individual emotions disgust (ASD group 60.5%; HC group: 72.6%; Δ = − 12.13 [90% CI − 25.05 to 0.79]; f^*2*^ = 0.052*; p* = 0.12; t = − 1.57; df = 48), and happiness (ASD group 51.7%; HC group: 62.5%; Δ = − 10.80 [90% CI − 22.11 to 0.52]; f^2^ = *0.05; p* = 0.12; t = − 1.6; df = 51). Interestingly, Participants with ASD identified the emotions fearful (ASD group 57.5%; HC 45.7%; Δ = 11.80 [90% CI –0.24 to 23.85]; f^2^ = *0.056; p* = 0.11; t = 1.64; df = 48) and surprise (ASD group 63.9%; HC group: 54.2%; Δ = 9.77 [90% CI − 0.82 to 20.36]; f^2^ = *0.048; p* = 0.13; t = 1.55; df = 50) more easily than HCs. However, none of these differences were statistically significant.

#### Olfaction

We first assessed the difference in smell identification scores between the two groups. No differences were observed between participants with ASD and HCs in the mean percentage of correct answers on the Sniffin’ Sticks Screening 12 olfaction identification test (ASD group: 84.8%; HC group: 90.2%; Δ = − 5.35 [90% CI − 13.06 to 2.37]; f^2^ = 0.04*; p* = 0.25; t = − 1.17; df = 34). Next, as a *post-hoc* analysis, we explored the potential of olfaction as a stratification factor by grouping participants according to their olfactory status. The olfactory status was defined by a threshold of 10 points on the Sniffin’ Sticks Screening 12 olfaction identification test based on normative data (Kobal et al. 1996). Seventeen HCs and 22 ASD participants were classified as normosmic; two HC and 16 ASD participants as hyposmic and anosmic. For this analysis, the two hyposmic HCs were excluded, since a sample size of two was not large enough to allow reliable comparisons with the other three groups [HC normosmic, ASD normosmic, ASD hyposmic]. Compared with HCs, we observed a significant higher rate of ASD participants with impaired olfaction (Fisher’s exact test: ASD 42.1%; HC 10.5%; *p* = 0.018; 95% CI [1.16–61.04]; sample estimates: OR 6.01). Impaired olfaction also identified meaningful subgroups in terms of IQ, adaptive skills, social functioning, and social cognition (Table 4).

**Table 4:** Differences between normal and impaired olfaction ASD groups

Variable	ASD normosmic (n = 22)	ASD hyposmic (n = 16)	Estimate normosmic versus hyposmic	*p* value	Cohen’s f^2^
WASI-II FS IQ	105.58	96.57	− 9.01	0.12	0.111
WASI-II VIQ	108.58	94.00	− 14.58	0.02	0.026
WASI-II PIQ	101.25	99.14	− 2.11	0.74	0.227
AQ total	29.08	27.57	− 1.51	0.63	0.011
STAI state/trait anxiety	45.58	43.57	− 2.01	0.74	0.008
ADOS communication	2.67	2.86	0.19	0.60	0.187
ADOS social interaction	6.75	7.29	0.54	0.56	0.006
ADOS communication and social interaction	9.42	10.00	0.58	0.59	0.118
ADOS total	12.00	13.29	1.29	0.30	0.168
VABS-II composite	62.83	67.00	4.17	0.44	0.043
VABS-II communication	61.67	68.00	6.33	0.36	0.063
VABS-II daily living	70.67	73.43	2.76	0.68	0.016
VABS-II socialization	62.58	66.00	3.42	0.61	0.012
ABC total	34.67	46.00	11.33	0.26	0.003
ABC irritability	5.75	12.71	6.96	0.05	0.049
ABC lethargy	14.25	14.43	0.18	0.96	0.038
ABC stereotypy	3.75	4.43	0.68	0.76	0.024
ABC hyperactivity/noncompliance	8.33	11.00	2.67	0.44	0.001
ABC inappropriate speech	2.58	3.43	0.85	0.53	0.103
ASR % correct	59.55	44.64	− 14.91	0.008	0.367
RMET % correct	71.06	51.19	− 19.87	0.006	0.381

Estimate refers to the estimated mean difference between normosmic and hyposmic participants, derived from an analysis of variance model. ASD hyposmic includes participants meeting both hyposmic and anosmic thresholds. P-values less than 0.00094 are considered statistically significant after multiplicity adjustment

*ABC* Aberrant behavior checklist, *ADOS* autism diagnostic observation schedule, *ASD* autism spectrum disorder, *AQ* autism quotient, *ASR* affective speech recognition, *FSIQ* full-scale intelligence quotient, *PIQ* performance intelligence quotient, *RMET* reading the mind in the eyes test-revised, *STAI* state/trait anxiety inventory, *VABS-II* Vineland Adaptive Behavior Scale-II, *VIQ* verbal intelligence quotient, *WASI-II* Wechsler Abbreviated Scale of Intelligence version II

Finally, we evaluated the association between olfactory status and the outcomes on the different assessments. When compared with participants with ASD with normal olfaction, the ASD participants with olfaction dysfunction showed reduced emotion recognition ability on two tasks of ToM: lower accuracy on the RMET (Δ = − 19.87 [90% CI − 31.29 to − 8.46]; f^2^ = 0.38; *p* < 0.01; t = − 2.54; df = 17) and on the ASR overall (Δ = − 14.91 [90% CI − 23.88 to − 5.93]; f^2^ = 0.367*; p* < 0.01; t = − 3.58; df = 35) driven mainly by a lower identification of the individual emotions -disgust (Δ = − 31.35 [90% CI − 48.22 to − 14.48]; *p* = 0.004) and surprise (Δ = − 18.65 [90% CI − 33.30 to − 4.01]; *p* = 0.039). The normosmic ASD subgroup showed performances comparable to those of the normosmic HCs on the percentage correct scores of ASR (Δ = 1.48 [90% CI − 5.64 to 8.40]; *p* = 0.73) and RMET (Δ = 7.86 [90% CI − 1.20 to 16.91]; *p* = 0.151). ASD participants with impaired olfaction showed a lower FSIQ (Δ = − 9.01 [90% CI − 18.63 to 0.60]; f^2^ = 0.111; *p* = 0.12; t = − 2.0; df = 36) influenced by a lower verbal IQ (Δ = − 14.58 [90% CI − 24.56 to − 4.60]; f^2^ = 0.026; *p* = 0.02; t = − 0.97; df = 36), and more irritability (ABC irritability subscale) (Δ = 6.96 [90% CI 1.12 to 12.81]; f^2^ = 0.049; *p* = 0.05; t = 1.33; df = 36) (Table 4) when compared with ASD participants with normal olfaction. Olfactory impairment did not influence the performance on eye tracking variables, VABS-II, or ADOS subdomain scores.

## Discussion

This study sought to identify discriminant properties of putative surrogate markers relating to social dysfunction in adults with ASD. Measures differentiating participants with ASD from HCs were pupillometry, quantifying arousal during task performance, and three of seven eye tracking paradigms, (preference for heads in activity monitoring, preference for biological motion compared with synthetic movements, and preference for videos of human movements compared with geometric shape videos). However, looking at the head during the activity monitoring task of the eye tracking was the only measures that survived Bonferroni correction, and no group difference in pupil size remained significant. Our findings are consistent with the majority of ASD literature, which relies heavily on studies of younger subjects. Our report is unique in that a few studies have applied such an extensive and broad battery of potential surrogate markers of ASD in adults, with the potential exception of consortia focused on this topic (EU-AIMS) (Loth et al. 2017), The Autism Biomarkers Consortium for Clinical Trials (ABC-CT), (Foundation for the National Institues of Health 2018), InFoR-Autism) (Fondation Fondamental 2018) and industry efforts i.e. JAKE® (Ness et al. 2017). One of the most novel findings relates to our identification of a high proportion of adults with ASD with evidence of impaired olfaction. Although it could be argued that the data may be skewed, since there appears to be a ceiling effect in the HC more than the ASD group, this 'compresses' the normal score. Hence a difference to an overall population of ASD displaying a larger variability is more difficult to demonstrate. A dichotomization by the olfactory status offers a solution. Among the general population, the prevalence of olfactory impairments seems to be age-related and has been reported to be between 19 and 22% in individuals between 16 and 55 years of age (Bramerson et al. 2004; Hummel et al. 2007; Vennemann et al. 2008). Doubling the normal rates, 42% of the participants with ASD showed olfaction dysfunction in our study. Despite small samples, differing olfaction test procedures, and non-standard scoring, it is notable that impairments in identification of odorants as well as differences in the rating of intensity and pleasantness/unpleasantness have been reported in adults with Asperger’s syndrome (Suzuki et al. 2003) and ASD (Wicker et al. 2016). Nevertheless, negative results have been reported as well: One used fewer stimuli and did not score per convention (Addo et al. 2017). Another study identified no differences in olfaction detection thresholds or adaptation to continued stimulus presentation in adults with ASD but did not test for accuracy (Tavassoli and Baron-Cohen 2012). However, our results were confirmed in the recently completed phase 2 study VANILLA (NCT01793441) (data on file, Roche) (Bolognani et al. 2019) in which the same olfaction test was assessed at baseline in 191 high-functioning male adults with ASD and 48.17% showed olfaction dysfunction. Taken together, our data coupled with prior reports provides significant support for an increased prevalence of olfaction dysfunction in ASD.

There is an increasing recognition that olfactory problems may be predictive of social impairment in children with ASD (Kumazaki et al. 2018; Lane et al. 2010; Hilton et al. 2007). Olfaction identification scores have been moderately correlated with reciprocal conversation skills (r = − 0.56) and social chatting scores (r = − 0.44) from the Autism Diagnostic Interview-Revised test (Bennetto et al. 2007) and taste/smell sensitivity has been identified as a predictor of maladaptive behaviors (r = − 0.53) measured by the VABS (Lane et al. 2010). Rozenkrantz et al., observed a significant association between sniff response to odor valence and the social affect component of the ADOS in children, together with an association between olfaction and FSIQ, thereby suggesting a mechanistic link between the response to olfactory stimuli and ASD through impaired sensory-motor systems that modulate social communication (Rozenkrantz et al. 2015). In the assessment of concurrent validity of this study, we observed that reduced olfaction was associated with worse emotion recognition ability on both RMET (r = 0.54) and ASR (r = 0.40), possibly indicating greater impairments in their TOM capacity, as well as communication deficits in the ADOS communication domain (r =  − 0.34) and in the inappropriate speech subscale of the ABC (r = − 0.32). Olfaction identification also correlated with the VIQ (r = 0.47) and FSIQ (r = 0.40) (Del Valle Rubido et al. 2018). These subanalyses did not control for differences in IQ, thus, the contribution of IQ differences to these associations is unknown. However, given the links between olfaction and the development of social cognition and the fact that olfactory identification also relies upon intact orbitofrontal cortical (OFC) functioning, further research is warranted to clarify both the potential of olfaction as a biomarker for social deficits in ASD and the underlying biological mechanisms.

Our findings are not surprising, as olfaction has been established as a critical element in affective matching after the age of 5 years in typically developing children (Cavazzana et al. 2016). It plays a key role in bonding (Bowlby 1980; Sullivan et al. 2011; Wedekind and Penn 2000) and highly influences interpersonal relationships (Huttenbrink et al. 2013). Research has already identified olfaction as an indicator of neuronal, social and cognitive development (Rozenkrantz et al. 2015), and it may also be a marker for severe central nervous pathology affecting social communication (Amaral et al. 2008; Huttenbrink et al. 2013). Krajnik et al. suggested a relationship between olfactory dysfunction and interoceptive awareness (Krajnik et al. 2015). Recent research has also drawn attention to the association between interoceptive abnormalities and ASD (Barttfeld et al. 2012; Elwin et al. 2012; Fiene and Brownlow 2015; Garfinkel et al. 2016; Hatfield et al. 2017; Noel et al. 2018) as well as other psychiatric disorders characterized by emotional impairment (Furman et al. 2013; Pollatos et al. 2009; Stevens et al. 2011). Interoception could also be associated with the sensory processing abnormalities found in ASD which are now an important aspect of the ASD diagnosis criteria per the Diagnostic and Statistical Manual of Mental Disorders, 5th Edition (American Psychiatric Association 2013). Correlations between olfactory dysfunction, sensory processing and interoception in ASD remain yet to be further elucidated.

Notably, another consistent finding were the larger pupil sizes during the eye-tracking assessments in the ASD group compared with the HC group, with moderate to large effect sizes (0.60 to 0.85) suggesting a dysregulated autonomic arousal in response to environmental stimulus as a prominent phenotype in ASD (Anderson and Colombo 2009; Kushki et al. 2013; Hirstein et al. 2001; Anderson et al. 2013; Corbett et al. 2010). We did not assess pupillometry using standardized stimuli (e.g. flashes of light, as seen in (Nystrom et al. 2015)) or baseline pupil measurements outside of the eye tracking experiments. In addition, this study was not designed to test pupil response but rather to provide a straightforward comparison of pupil sizes during tasks. Therefore, it is unknown whether the larger pupil sizes are a baseline characteristic, a reaction to the task or to specific social or non-social stimuli within each task. Increased tonic pupillary size noted in children with ASD with evidence of lower sympathetic tone, (Anderson et al. 2013), and lower electrodermal activity and responses (Kushki et al. 2013) support the position of abnormal autonomic nervous system response in pathophysiology of ASD. In our correlation analysis of these measures, pupillometry was mostly unrelated to ASD severity and core social deficits, except for the biomotion task that correlated with the ADOS total score (r = − 0.33) and the communication subdomain of the Vineland (r = 0.36). However, larger pupil size was consistently related to lower behavioral ratings of hyperactivity on the ABC (r values ranging from − 0.36 to − 0.43) and higher FSIQ (ranging from 0.38 to 0.45) and PIQ scores (ranging from 0.36 to 0.44) while the VIQ remained unrelated (Del Valle Rubido et al. 2018). Thus, in ASD, better functioning is associated with larger pupil sizes.

Pupil dilation is known to be modulated by the brain’s locus coeruleus-norepinephrine system (Rajkowski et al. 1993), which controls physiological arousal (Samuels and Szabadi 2008) and cognitive functioning (Ramos and Arnsten 2007; Sara 2009) and has been used as a measure of subjective task difficulty, mental effort, and neural gain (Eckstein et al 2017). As a reflection of greater arousal or effort while engaged in task performance, pupil size may indicate the ability to better marshal effortful attention during the eye tracking as a sign of greater cognitive or inhibitory control and prove its utility in studying this separate important dimension of co‐occurring inattentive and disruptive behavior symptoms in ASD (McCracken 2011) or intellectual disability. While this explanation would appear to be inconsistent with the between-group difference observed, where ASD participants were shown to have larger pupil sizes than HCs, it is important to note that all eye-tracking tasks presented were fundamentally implicit or explicit tasks of social cognition. It is possible that HCs required less effortful attention to complete these tasks due to an inherently greater facility in social information processing. Another possibility is a proposed model of chronic autonomic nervous system hyperarousal in ASD, which describes chronic biological threat response, forwarded by Patriquin et al*.* based on a review of cardiac literature in adults and children with ASD (Patriquin et al., 2019; Edmiston et al. 2016; Guy et al. 2014; Bal et al. 2010; Van Hecke et al. 2009; Ming et al. 2005; Denver 2004). Based on the Polyvagal Theory (Porges 1995), Patriquin et al*.* suggest a potential difference of the information flowing from the brain to periphery in individuals with ASD due to differences in the neuroception of safety versus threat, resulting in greater autonomic hyperarousal in ASD. Latent hyperarousal differences between ASD and HCs could explain between-group pupil size differences observed, with this effect modulated by differences in autonomic flexibility observed between individuals with ASD with and without intellectual impairment (Van Hecke et al. 2009; Cohen and Johnson 1977; Goodwin et al. 2006; Miller and Bernal 1971; Palkovitz and Wiesenfeld 1980; Sigman et al. 2003; Sheinkopf et al. 2013). Although, the precise determinants of increased pupillary size in ASD remain to be clarified, pupillometry could also be informative for subject stratification efforts, depending on intervention.

Extending the results of previous work, we demonstrated atypical gaze patterns in eye tracking in the activity monitoring, biological motion preference and human preference tasks (Annaz et al. 2012; Chawarska et al. 2013; Frederick Shic et al. 2014) in the ASD group. However, no differences were observed in the remaining four out of seven eye-tracking paradigms (biodetection, WAVW, gaze discrimination and gender discrimination). This contrasts with results of many previous studies in younger subjects which showed that the best predictor of autism was reduced eye region fixation time (Auyeung et al. 2015; Klin et al. 2002). Moreover, despite the association found between looking at the mouth and social communication skills (Del Valle Rubido et al. 2018), there was no difference in fixation in the mouth between groups.

The failure to replicate previous eye tracking findings may be explained by several factors: firstly, potential under-reporting of negative and inconclusive results, because of the dearth of studies investigating eye pattern differences in adults with and without ASD or subgroups within the ASD population (Zamzow et al. 2014); divergent eye gaze patterns may depend on the nature of the stimuli presented (dynamic or static, real-life and naturalistic or non-naturalistic, social or non-social) (Hanley et al. 2015; Hanley et al. 2013; Speer et al. 2007; Manyakov et al 2018). More likely, however, is the possibility that high-functioning adults with ASD might ultimately succeed in reaching the developmental level of neurotypicals with overall minor differences in eye gaze patterns (Baez et al. 2012; Ullman and Pullman 2015) by developing compensatory mechanisms, or implementation of strategies to read faces (Bauminger 2002; Dawson et al. 2005; Hwang and Hughes 2000) and/or detect biological motion.

Our study also showed little relationship between eye tracking measures, adaptive behaviors measured by the Vineland, other measures of social perception and olfaction. Nonetheless, small to moderate correlations were found between activity monitoring, WAVW, and gender discrimination tasks with the severity of ASD symptoms and behavior measured by the ADOS and ABC (Del Valle Rubido et al 2018). Of all these tasks, the only paradigm for which there were consistent findings between correlation results (Del Valle Rubido et al. 2018) and the between-group differences highlighted here were in looking at the people in Activity Monitoring (greater looking at people associated with lower autism symptom severity in ASD, and less looking at people, especially the head, in ASD as compared to HCs). Associations with phenotype within ASD and ASD-HC between group differences were in an opposite-to-expected direction for human activity preference, with poorer adaptive communication associated with greater human looking within ASD, but less looking at the human versus geometric shape observed here in ASD as compared to HC. Other tasks showed significant findings for one of either correlations (Del Valle Rubido et al 2018) or between-group comparisons, but not both. These patterns highlight the complexity of straightforward extensions of between-group comparisons of ASD and HC groups to relationships within ASD. Factors which may impact the directionality and strength of effects could include reduced dynamic range within the ASD or HC groups, comorbid psychiatric features such as anxiety or depression in ASD, as well as fundamentally different mechanisms impacting social scene gaze patterns within ASD as compared to across groups, similar to that for which we have forwarded for pupil size relationships. Further studies are necessary to clarify these complex relationships.

Perhaps somewhat surprising was the lack of group differences observed between the ASD and HC groups for two measures, the RMET and the ASR, contrary to prior studies (Baron-Cohen et al. 2001a, b, 2015; Holt et al. 2014; Kaland et al. 2008). A review by Sivaratnam et al. found inconsistent reports of ToM impairments in structured test settings in high-functioning ASD groups (Happe 1995; Bauminger 2002), in contrast to clear impairments revealed in naturalistic test settings (Rump et al. 2009; Dziobek et al. 2006) and in everyday functioning (Rieffe et al. 2000). Suggesting that paradigms measuring ToM in non-naturalistic social settings may not provide an accurate pattern of functioning in ASD groups (Sivaratnam et al. 2015; Adolphs 2001; Klin 2000; Baron-Cohen et al. 1985; Leslie and Frith 1990; Weeks and Hobson 1987). Klin et al. (2003) theorized that due to the differences in learning, individuals with ASD may develop compensatory strategies which help them score well on standardized tests. Yet, difficulties may remain when the applying the cognitive potential and the appropriate set of social skills in naturalistic contexts (Klin et al. 2007, 2003). Our findings also reflect this contradiction. On the one hand, despite the lack of group differences, both the ASR and RMET demonstrated significant relationships with each other (r = − 0.64) but neither did they correlate with the ADOS communication and reciprocal social interaction domains. On the other hand, both the ASR and RMET correlated with the Inappropriate Speech subscale of the ABC (ASR r = − 0.66, RMET r = − 0.52) and the ASR with the Vineland communication subdomain and the adaptive behavior composite score (r = 0.46 and r = 0.40 respectively). It remains unclear whether the lack of group differences despite existing correlations between the ASR and RMET and the Vineland and ABC is due to the non-naturalistic test setting. In addition, the difference in how the concepts of socialization and communication are measured with the various clinical assessments (symptomatology/ disability in ADOS vs. ability in Vineland (Klin et al. 2007) and problematic behaviors in ABC) could be an additional confounding factor to be taken into consideration. When looking at the individual emotions in the ASR, ASD participants did not identify disgust and happiness as easily as healthy controls, whereas they were able to identify fearfulness and surprise. This over-responsiveness for fearfulness and surprise observed in with the ASD group, is perhaps indicative of higher levels of anxiety or a lack of understanding and inappropriate expression of emotions in ASD (Shields et al. 1994; Sigman et al. 1992). A plausible mechanism for the higher level of anxiety could be an increased activation of subcortical brain regions (i.e., amygdala) involved in the processing of fearful faces differs in subjects with ASD compared with HCs in functional magnetic resonance imaging (Kleinhans et al. 2015, 2011). These findings in ToM warrant further research to understand the underlying mechanisms. The higher level of complexity and effort required of both the RMET and ASR compared to passive viewing of faces in the eye tracking and pupillometry, may have led to the lack of differences.

## Limitations

The included studies both enrolled a relatively small sample size of all-male, high-functioning adults, limiting the generalizability of these findings. Participants with ASD were required to have ABC irritability subscale scores ≤ 13 and to undergo a 2-h infusion in Study 2, which may further limit applicability to lower-functioning, more severe and disruptive ASD phenotypes. The time required to complete the assessments was long (8 h), which may have created substantial burden of cognitive load on the participants. It can also be argued that the ASD and HC groups, while matched on age, were not well matched on other potential confounding factors. For instance, the race characteristics of the participants in ASD and HC groups were different i.e., a preponderance of Caucasian participants in the ASD group) and socioeconomic aspects were not considered. These fundamental differences between groups limit the applicability of these results to broader ASD populations. The analyses performed are exploratory. These include the *post-hoc* analysis of the ASD population by olfaction status based on the odor identification subtest of the Sniffin’ Sticks Screening 12 olfaction identification test. As such, between-group differences and *p*-values should be interpreted with caution and used as a guidance for selection of assessments in future studies. Finally, despite the selection of Screening 12 version of the Sniffin’ sticks test for its convenient administration in everyday clinical practice, other versions of the Sniffin’ Sticks test may allow a more precise testing and therefore an improved characterization of olfactory performances.

## Conclusions

Our results suggest a potential use of specific eye tracking tasks, pupillometry and olfaction tests for stratification and response sub-analyses outcome-prediction in ASD trials. They also highlight the fact that abnormalities reported in young individuals with ASD may no longer be present to the same extent or with the same profile in adults with ASD. This points towards the view that the profile of abnormalities and hence characteristics of potential markers may change with development. The eye tracking, activity monitoring, biological motion, human activity preference and pupillometry tasks differentiated the best between paticipants with ASD and HCs. Our results implicate olfaction as a factor in the development of social cognition. It may be a simple and useful assessment for characterization of disease severity and for stratification in clinical trials. However, replication is needed for confirmatory purpose, and additional research should clarify sensitivity to change and links to functional outcomes.

## Electronic supplementary material

Below is the link to the electronic supplementary material.

Supplementary file1 (DOCX 27 kb)

Supplementary file2 (PDF 401 kb)

## Data Availability

Qualified researchers may request access to individual patient level data through the clinical study data request platform (www.clinicalstudydatarequest.com). Further details on Roche's criteria for eligible studies are available here (https://clinicalstudydatarequest.com/Study-Sponsors/Study-Sponsors-Roche.aspx). For further details on Roche's Global Policy on the Sharing of Clinical Information and how to request access to related clinical study documents, see here (https://www.roche.com/research_and_development/who_we_are_how_we_work/clinical_trials/our_commitment_to_data_sharing.htm).

## References

[CR1] Addo RN, Wiens S, Nord M, Larsson M (2017). Olfactory functions in adults with Autism Spectrum Disorders. Perception.

[CR2] Adolphs R (2001). The neurobiology of social cognition. Current Opinion in Neurobiology.

[CR3] Aman MG, Singh NN, Stewart AW, Field CJ (1985). Psychometric characteristics of the aberrant behavior checklist. American Journal of Mental Deficiency.

[CR4] Amaral DG, Schumann CM, Nordahl CW (2008). Neuroanatomy of autism. Trends in Neurosciences.

[CR5] American Psychiatric Association (2000). Diagnostic and statistical manual-text revision (DSM-IV-TRim, 2000).

[CR6] American Psychiatric Association (2013). Diagnostic and statistical manual of mental disorders, fifth edition (DSM-5).

[CR7] Anagnostou E, Jones N, Huerta M, Halladay AK, Wang P, Scahill L (2015). Measuring social communication behaviors as a treatment endpoint in individuals with autism spectrum disorder. Autism.

[CR8] Andari E, Duhamel J-R, Zalla T, Herbrecht E, Leboyer M, Sirigu A (2010). Promoting social behavior with oxytocin in high-functioning autism spectrum disorders. Proceedings of the National Academy of Sciences.

[CR9] Anderson CJ, Colombo J (2009). Larger tonic pupil size in young children with Autism Spectrum Disorder. Developmental Psychobiology.

[CR10] Anderson CJ, Colombo J, Unruh KE (2013). Pupil and salivary indicators of autonomic dysfunction in autism spectrum disorder. Developmental Psychobiology.

[CR11] Annaz D, Campbell R, Coleman M, Milne E, Swettenham J (2012). Young children with autism spectrum disorder do not preferentially attend to biological motion. Journal of Autism and Developmental Disorders.

[CR12] Auyeung B, Lombardo MV, Heinrichs M, Chakrabarti B, Sule A, Deakin JB (2015). Oxytocin increases eye contact during a real-time, naturalistic social interaction in males with and without autism. Translational Psychiatry.

[CR13] Baez S, Rattazzi A, Gonzalez-Gadea ML, Torralva T, Vigliecca NS, Decety J (2012). Integrating intention and context: assessing social cognition in adults with Asperger syndrome. Frontiers in Human Neuroscience.

[CR14] Baio J, Wiggins L, Christensen DL, Maenner MJ, Daniels J, Warren Z (2018). Prevalence of autism spectrum disorder among children aged 8 years—autism and developmental disabilities monitoring network, 11 sites, United States, 2014. MMWR Surveill Summ.

[CR15] Bal E, Harden E, Lamb D, Van Hecke AV, Denver JW, Porges SW (2010). Emotion recognition in children with autism spectrum disorders: Relations to eye gaze and autonomic state. Journal of Autism and Developmental Disorders.

[CR16] Baron-Cohen S, Leslie AM, Frith U (1985). Does the autistic child have a “theory of mind”?. Cognition.

[CR17] Baron-Cohen W, Wheelwright S, Hill J, Raste Y, Plumb I (2001). The “Reading the Mind in the Eyes” test revised version: A study with normal adults, and adults with Asperger syndrome or high-functioning autism. Journal of Child Psychology and Psychiatry.

[CR18] Baron-Cohen S, Jolliffe T, Mortimore C, Robertson M (1997). Another advanced test of theory of mind: Evidence from very high functioning adults with autism or Asperger syndrome. Journal of Child Psychology and Psychiatry.

[CR19] Baron-Cohen S, Wheelwright S, Skinner R, Martin J, Clubley E (2001). The autism-spectrum quotient (AQ): Evidence from Asperger syndrome/high-functioning autism, males and females, scientists and mathematicians. Journal of Autism and Developmental Disorders.

[CR20] Baron-Cohen B, Daniel C, Holt RJ, Allison C, Auyeung B, Lombardo MV, Smith P (2015). The “Reading the mind in the eyes” test: Complete absence of typical sex difference in ~400 men and women with autism. PLoS ONE.

[CR21] Barttfeld P, Wicker B, Cukier S, Navarta S, Lew S, Leiguarda R (2012). State-dependent changes of connectivity patterns and functional brain network topology in autism spectrum disorder. Neuropsychologia.

[CR22] Bauminger N (2002). The facilitation of social-emotional understanding and social interaction in high-functioning children with autism: Intervention outcomes. Journal of Autism and Developmental Disorders.

[CR23] Baxter AJ, Brugha TS, Erskine HE, Scheurer RW, Vos T, Scott JG (2015). The epidemiology and global burden of autism spectrum disorders. Psychological Medicine.

[CR24] Bennetto L, Kuschner ES, Hyman SL (2007). Olfaction and taste processing in autism. Biological Psychiatry.

[CR25] Bolognani F, Del Valle Rubido M, Squassante L, Wandel C, Derks M, Murtagh L, Sevigny J, Kwhaja O, Fontoura P (2019). A Phase 2 clinical trial of a vasopressin V1a receptor antagonist shows improved adaptive behaviors in men with autism spectrum disorder. Science Translational Medicine.

[CR26] Bowlby J (1980). Attachment and loss.

[CR27] Bramerson A, Johansson L, Ek L, Nordin S, Bende M (2004). Prevalence of olfactory dysfunction: The skovde population-based study. Laryngoscope.

[CR28] Brugha TS, Doos L, Tempier A, Einfeld S, Howlin P (2015). Outcome measures in intervention trials for adults with autism spectrum disorders; A systematic review of assessments of core autism features and associated emotional and behavioural problems. International Journal of Methods in Psychiatric Research.

[CR29] Carnegie Mellon University Graphics Lab CMU Graphics Lab motion capture database. Retrieved September 06, 2011, from https://mocap.cs.cmu.edu/.

[CR30] Cavazzana A, Wesarg C, Parish-Morris J, Lundstrom JN, Parma V (2016). When preschoolers follow their eyes and older children follow their noses: Visuo-olfactory social affective matching in childhood. Developmental Science.

[CR31] Charman T, Loth E, Tillmann J, Crawley D, Wooldridge C, Goyard D (2017). The EU-AIMS Longitudinal European Autism Project (LEAP): Clinical characterisation. Molecular Autism.

[CR32] Chawarska K, Macari S, Shic F (2013). Decreased spontaneous attention to social scenes in 6-month-old infants later diagnosed with Autism Spectrum Disorders. Biological Psychiatry.

[CR33] Chita-Tegmark M (2016). Social attention in ASD: A review and meta-analysis of eye-tracking studies. Research In Developmental Disabilities.

[CR34] Cohen DJ, Johnson WT (1977). Cardiovascular correlates of attention in normal and psychiatrically disturbed children: Blood pressure, peripheral blood flow, and peripheral vascular resistance. Archives of General Psychiatry.

[CR36] Corbett BA, Schupp CW, Simon D, Ryan N, Mendoza S (2010). Elevated cortisol during play is associated with age and social engagement in children with autism. Molecular Autism.

[CR37] Dawson G, Webb SJ, McPartland J (2005). Understanding the nature of face processing impairment in autism: Insights from behavioral and electrophysiological studies. Developmental Neuropsychology.

[CR38] Del Valle Rubido M, McCracken JT, Hollander E, Shic F, Noeldeke J, Boak L (2018). In search of biomarkers for autism spectrum disorder. Autism Research.

[CR39] Denver, J. W. (2004). The social engagement system: Functional differences in individuals with autism. Retrieved from https://drum.lib.umd.edu/bitstream/1903/1351/1/umi-umd-1486.pdf.

[CR40] Dziobek I, Fleck S, Kalbe E, Rogers K, Hassenstab J, Brand M, Convit A (2006). Introducing MASC: A movie for the assessment of social cognition. Journal of Autism and Developmental Disorders.

[CR41] Eckstein MK, Guerra-Carrillo B, Miller Singley AT, Bunge SA (2017). Beyond eye gaze: What else can eyetracking reveal about cognition and cognitive development?. Developmental Cognitive Neuroscience.

[CR42] Edmiston EK, Jones RM, Corbett BA (2016). Physiological response to social evaluative threat in adolescents with autism spectrum disorder. Journal of Autism and Developmental Disorders.

[CR43] Elwin M, Ek L, Schroder A, Kjellin L (2012). Autobiographical accounts of sensing in Asperger syndrome and high-functioning autism. Archives of Psychiatric Nursing.

[CR44] Endevelt-Shapira Y, Perl O, Ravia A, Amir D, Eisen A, Bezalel V (2018). Altered responses to social chemosignals in autism spectrum disorder. Nature Neuroscience.

[CR45] Fiene L, Brownlow C (2015). Investigating interoception and body awareness in adults with and without autism spectrum disorder. Autism Research.

[CR46] Fondation Fondamental. (2018). INFOR-AUTISM. Retrieved Septemner 05, 2018, from https://www.fondation-fondamental.org/avancer-avec-la-recherche/les-projets-de-fondamental/infor-autism.

[CR47] Foundation for the National Institues of Health. (2018). Biomarkers Consortium—The Autism Biomarkers Consortium for Clinical Trials (ABC-CT). Retrieved September 05, 2018, from https://fnih.org/what-we-do/biomarkers-consortium/programs/autism-biomarkers.

[CR48] Frazier TW, Strauss M, Klingemier EW, Zetzer EE, Hardan AY, Eng C (2017). A meta-analysis of gaze differences to social and nonsocial information between individuals with and without autism. Journal of the American Academy of Child and Adolescent Psychiatry.

[CR49] Furman DJ, Waugh CE, Bhattacharjee K, Thompson RJ, Gotlib IH (2013). Interoceptive awareness, positive affect, and decision making in major depressive disorder. Journal of Affective Disorders.

[CR50] Garfinkel SN, Tiley C, O'Keeffe S, Harrison NA, Seth AK, Critchley HD (2016). Discrepancies between dimensions of interoception in autism: Implications for emotion and anxiety. Biological Psychology.

[CR51] Goodwin MS, Groden J, Velicer WF, Lipsitt LP, Baron MG, Hofmann SG (2006). Cardiovascular arousal in individuals with autism. Focus on Autism and Other Developmental Disabilities.

[CR52] Guy L, Souders M, Bradstreet L, DeLussey C, Herrington JD (2014). Brief report: Emotion regulation and respiratory sinus arrhythmia in autism spectrum disorder. Journal of Autism and Developmental Disorders.

[CR53] Guy, W. (1976). ECDEU assessment manual for psychopharmacology. Revised National Institute of Mental Health

[CR54] Hanley M, McPhillips M, Mulhern G, Riby DM (2013). Spontaneous attention to faces in Asperger syndrome using ecologically valid static stimuli. Autism.

[CR55] Hanley M, Riby DM, Carty C, McAteer AM, Kennedy A, McPhillips M (2015). The use of eye-tracking to explore social difficulties in cognitively able students with autism spectrum disorder: A pilot investigation. Autism.

[CR56] Hatfield TR, Brown RF, Giummarra MJ, Lenggenhager B (2017). Autism spectrum disorder and interoception: Abnormalities in global integration?. Autism.

[CR57] Happe FG (1995). The role of age and verbal ability in the theory of mind task performance of subjects with autism. Child Development.

[CR58] Hays WST (2003). Human pheromones: Have they been demonstrated?. Behavioral Ecology and Sociobiology.

[CR59] Heilman KM, Scholes R, Watson RT (1975). Auditory affective agnosia. Disturbed comprehension of affective speech. Journal of Neurology, Neurosurgery, and Psychiatry.

[CR60] Hilton C, Graver K, LaVesser P (2007). Relationship between social competence and sensory processing in children with high functioning autism spectrum disorders. Research in Autism Spectrum Disorders.

[CR61] Hirstein W, Iversen P, Ramachandran VS (2001). Autonomic responses of autistic children to people and objects. Proceedings Biological Sciences.

[CR62] Hollander E, Bartz J, Chaplin W, Phillips A, Sumner J, Soorya L (2007). Oxytocin increases retention of social cognition in autism. Biological Psychiatry.

[CR63] Holt RJ, Chura LR, Lai M-C, Suckling J, von dem Hagen E, Calder AJ (2014). ‘Reading the mind in the eyes’: An fMRI study of adolescents with autism and their siblings. Psychological Medicine.

[CR64] Hummel T, Kobal G, Gudziol H, Mackay-Sim A (2007). Normative data for the “Sniffin’ Sticks” including tests of odor identification, odor discrimination, and olfactory thresholds: An upgrade based on a group of more than 3,000 subjects. European Archives of Oto-Rhino-Laryngology.

[CR65] Huttenbrink KB, Hummel T, Berg D, Gasser T, Hahner A (2013). Olfactory dysfunction: Common in later life and early warning of neurodegenerative disease. Deutsches Ärzteblatt International.

[CR66] Hwang B, Hughes C (2000). The effects of social interactive training on early social communicative skills of children with autism. Journal of Autism and Developmental Disorders.

[CR67] Jeste SS, Geschwind DH (2014). Disentangling the heterogeneity of autism spectrum disorder through genetic findings. Nature Reviews Neurology.

[CR68] Ji N, Findling R (2015). An update on pharmacotherapy for autism spectrum disorder in children and adolescents. Current Opinion in Psychiatry.

[CR69] Kaiser MD, Delmolino L, Tanaka JW, Shiffrar M (2010). Comparison of visual sensitivity to human and object motion in autism spectrum disorder. Autism Research.

[CR70] Kaland N, Callesen K, Moller-Nielsen A, Mortensen EL, Smith L (2008). Performance of children and adolescents with Asperger syndrome or high-functioning autism on advanced theory of mind tasks. Journal of Autism and Developmental Disorders.

[CR71] Kleinhans NM, Richards T, Greenson J, Dawson G, Aylward E (2015). Altered dynamics of the fMRI response to faces in individuals with autism. Journal of Autism and Developmental Disorders.

[CR72] Kleinhans NM, Richards T, Johnson LC, Weaver KE, Greenson J, Dawson G (2011). fMRI evidence of neural abnormalities in the subcortical face processing system in ASD. NeuroImage.

[CR73] Klin A (2000). Attributing social meaning to ambiguous visual stimuli in higher-functioning autism and Asperger syndrome: The social attribution task. Journal of Child Psychology and Psychiatry, and Allied Disciplines.

[CR74] Klin A, Jones W, Schultz R, Volkmar F, Cohen D (2002). Visual fixation patterns during viewing of naturalistic social situations as predictors of social competence in individuals with autism. Archives of General Psychiatry.

[CR75] Klin A, Jones W, Schultz R, Volkmar F (2003). The enactive mind, or from actions to cognition: Lessons from autism. Philosophical Transactions Biological Sciences.

[CR76] Klin A, Saulnier CA, Sparrow SS, Cicchetti DV, Volkmar FR, Lord C (2007). Social and communication abilities and disabilities in higher functioning individuals with autism spectrum disorders: the Vineland and the ADOS. Journal of Autism and Developmental Disorders.

[CR77] Kobal G, Hummel T, Sekinger B, Barz S, Roscher S, Wolf S (1996). "Sniffin' sticks": Screening of olfactory performance. Rhinology.

[CR78] Krajnik J, Kollndorfer K, Notter LA, Mueller CA, Schopf V (2015). The impact of olfactory dysfunction on interoceptive awareness. Psychophysiology.

[CR79] Kumazaki H, Okamoto M, Kanzaki S, Okada KI, Mimura M, Minabe Y (2018). Approaches for assessing olfaction in children with autism spectrum disorder. Methods in Molecular Biology.

[CR80] Kushki A, Drumm E, Pla Mobarak M, Tanel N, Dupuis A, Chau T (2013). Investigating the autonomic nervous system response to anxiety in children with autism spectrum disorders. PLoS ONE.

[CR81] Laeng B, Sirois S, Gredeback G (2012). Pupillometry: A window to the preconscious?. Perspectives on Psychological Science.

[CR82] Lai MC, Lombardo MV, Chakrabarti B, Baron-Cohen S (2013). Subgrouping the autism "spectrum": Reflections on DSM-5. PLoS Biology.

[CR83] Lane AE, Young RL, Baker AE, Angley MT (2010). Sensory processing subtypes in autism: Association with adaptive behavior. Journal of Autism and Developmental Disorders.

[CR84] Leslie AM, Frith U (1990). Prospects for a cognitive neuropsychology of autism: Hobson’s choice. Psychological Review.

[CR85] Lord C, Rutter M, DiLavore P, Risi S (2002). Autism diagnostic observation schedule: ADOS.

[CR86] Loth E, Charman T, Mason L, Tillmann J, Jones EJH, Wooldridge C, Ahmad J, Auyeung B, Brogna C, Ambrosino S, Banaschewski T, Baron-Cohen S, Baumeister S, Beckmann C, Brammer M, Brandeis D, Bölte S (2017). The EU-AIMS Longitudinal European Autism Project (LEAP): design and methodologies to identify and validate stratification biomarkers for autism spectrum disorders. Molecular Autism.

[CR87] Masi A, DeMayo MM, Glozier N, Guastella AJ (2017). An overview of autism spectrum disorder, heterogeneity and treatment options. Neuroscience Bullet.

[CR88] McCracken JT, Hollander E, Kolevzon A, Coyle JT (2011). Disruptive Behaviors. Textbook of autism spectrum disorders.

[CR89] Manyakov NV, Bangerter A, Chatterjee M, Mason L, Ness S, Lewin D, Skalkin A, Boice M, Goodwin MS, Dawson G, Hendren R, Leventhal B, Shic F, Pandina G (2018). Visual exploration in autism spectrum disorder: Exploring age differences and dynamic features using recurrence quantification analysi. Autism Research.

[CR90] Miller WH, Bernal ME (1971). Measurement of the cardiac response in schizophrenic and normal children. Psychophysiology.

[CR91] Ming X, Julu P, Brimacombe M, Connor S, Daniels M (2005). Reduced cardiac parasympathetic activity in children with autism. Brain & Development.

[CR150] Ness, S. L., Manyakov, N. V., Bangerter, A., Lewin, D., Jagannatha, S., Boice, M., et al. (2017). JAKE® Multimodal Data Capture System: Insights from an Observational Study of Autism Spectrum Disorder. *Front Neurosci,**11*, 517. 10.3389/fnins.2017.00517. 10.3389/fnins.2017.00517 PMC5623040 29018317

[CR92] Noel JP, Lytle M, Cascio C, Wallace MT (2018). Disrupted integration of exteroceptive and interoceptive signaling in autism spectrum disorder. Autism Res.

[CR93] Nystrom P, Gredeback G, Bolte S, Falck-Ytter T, Team E (2015). Hypersensitive pupillary light reflex in infants at risk for autism. Mol Autism.

[CR94] Ostrowski NL, Lolait SJ, Young WS (1994). Cellular localization of vasopressin V1a receptor messenger ribonucleic acid in adult male rat brain, pineal, and brain vasculature. Endocrinology.

[CR95] Palkovitz RJ, Wiesenfeld AR (1980). Differential autonomic responses of autistic and normal children. Journal of Autism and Developmental Disorders.

[CR96] Patriquin MA, Hartwig EM, Friedmand BH, Porgese SW, Scarpad A (2019). Autonomic response in autism spectrum disorder: Relationship to social and cognitive functioning. Biological Psychology.

[CR97] Pierce K, Conant D, Hazin R, Stoner R, Desmond J (2011). Preference for geometric patterns early in life as a risk factor for autism. Archives of General Psychiatry.

[CR98] Pierce K, Marinero S, Hazin R, McKenna B, Barnes CC, Malige A (2015). Eye tracking reveals abnormal visual preference for geometric images as an early biomarker of an autism spectrum disorder subtype associated with increased symptom severity. Biological Psychiatry.

[CR99] Pollatos O, Traut-Mattausch E, Schandry R (2009). Differential effects of anxiety and depression on interoceptive accuracy. Depress Anxiety.

[CR100] Porges SW (1995). Orienting in a defensive world: Mammalian modifications of our evolutionary heritage: A polyvagal theory. Psychophysiology.

[CR101] Rajkowski, J., Kubiak, P., Aston-Jones, G. (1993). Correlations between locuscoeruleus (LC) neural activity, pupil diameter and behavior in monkey supporta role of LC in attention. In *Society for Neuroscience Abstract. Presented at theSociety for Neuroscience Conference* (p. 974).

[CR102] Ramos BP, Arnsten AFT (2007). Adrenergic pharmacology and cognition: Focus on the prefrontal cortex. Pharmacology & Therapeutics.

[CR103] Rieffe C, Meerum Terwogt M, Stockmann L (2000). Understanding atypical emotions among children with autism. Journal of Autism and Developmental Disorders.

[CR104] Rozenkrantz L, Zachor D, Heller I, Plotkin A, Weissbrod A, Snitz K (2015). A mechanistic link between olfaction and autism spectrum disorder. Current Biology.

[CR105] Rump KM, Giovannelli JL, Minshew NJ, Strauss MS (2009). The development of emotion recognition in individuals with autism. Child Development.

[CR106] Sara SJ (2009). The locus coeruleus and noradrenergic modulation of cognition. Nature Reviews Neuroscience.

[CR107] Sarrett JC, Rommelfanger KS (2015). Commentary: Attention to eyes is present but in decline in 2–6-month-old infants later diagnosed with autism. Frontiers in Public Health.

[CR108] Samuels ER, Szabadi E (2008). Functional neuroanatomy of the noradrenergiclocus coeruleus: Its roles in the regulation of arousal and autonomic functionpart I: Principles of functional organisation. Current Neuropharmacology.

[CR109] Secundo L, Snitz K, Sobel N (2014). The perceptual logic of smell. Current Opinion in Neurobiology.

[CR110] Sheinkopf SJ, Neal-Beevers AR, Levine TP, Miller-Loncar C, Lester B (2013). Parasympathetic response profiles related to social functioning in young children with autistic disorder. Autism Research and Treatment.

[CR111] Shic, F. (2008). Computational methods for eye-tracking analysis: Applications to autism. Ph.D. thesis, Yale University.

[CR112] Shic F, Bradshaw J, Klin A, Scassellati B, Chawarska K (2011). Limited activity monitoring in toddlers with autism spectrum disorder. Brain Research.

[CR113] Shic F, Macari S, Chawarska K (2014). Speech disturbs face scanning in 6-month-old infants who develop autism spectrum disorder. Biological Psychiatry.

[CR114] Shields A, Cicchetti D, Ryan RM (1994). The development of emotional and behavioral self regulation and social competence among maltreated school-age children. Development and Psychopathology.

[CR115] Sigman MD, Kasari C, Kwon JH, Yirmiya N (1992). Responses to the negative emotions of others by autistic, mentally retarded, and normal children. Child Development.

[CR116] Sigman M, Dissanayake C, Corona R, Espinosa M (2003). Social and cardiac responses of young children with autism. Autism.

[CR117] Sivaratnam CS, Newman LK, Tonge BJ (2015). Attachment and emotion processing in children with autism spectrum disorders: Neurobiological, neuroendocrine, and neurocognitive considerations. Review Journal of Autism and Developmental Disorders.

[CR118] Sparrow S, Kreutzer J, DeLuca J, Caplan B (2011). Vineland Adaptive Behavior Scales. Encyclopedia of clinical neuropsychology.

[CR119] Speer LL, Cook AE, McMahon WM, Clark E (2007). Face processing in children with autism: Effects of stimulus contents and type. Autism.

[CR120] Spielberger CD (2010). State-trait anxiety inventory.

[CR121] Stevens S, Gerlach AL, Cludius B, Silkens A, Craske MG, Hermann C (2011). Heartbeat perception in social anxiety before and during speech anticipation. Behaviour Research and Therapy.

[CR122] Stevenson RJ (2010). An initial evaluation of the functions of human olfaction. Chemical Senses.

[CR123] Sullivan R, Perry R, Sloan A, Kleinhaus K, Burtchen N (2011). Infant bonding and attachment to the caregiver: Insights from basic and clinical science. Clinics in Perinatology.

[CR124] Suzuki Y, Critchley HD, Rowe A, Howlin P, Murphy DG (2003). Impaired olfactory identification in Asperger's syndrome. The Journal of Neuropsychiatry and Clinical Neurosciences.

[CR125] Tavassoli T, Baron-Cohen S (2012). Olfactory detection thresholds and adaptation in adults with autism spectrum condition. Journal of Autism and Developmental Disorders.

[CR126] Ullman MT, Pullman MY (2015). A compensatory role for declarative memory in neurodevelopmental disorders. Neuroscience & Biobehavioral Reviews.

[CR127] Umbricht D, del Valle Rubido M, Hollander E, McCracken J, Shic F, Scahill L (2016). A single dose, placebo-controlled proof-of-mechanism study of a novel vasopressin 1a receptor antagonist (RG7713) in high-functioning adult autism. Neuropsychopharmacology.

[CR128] Van Hecke AV, Lebow J, Bal E, Lamb D, Harden E, Kramer A (2009). Electroencephalogram and heart rate regulation to familiar and unfamiliar people in children with autism spectrum disorders. Child Development.

[CR129] Vennemann MM, Hummel T, Berger K (2008). The association between smoking and smell and taste impairment in the general population. Journal of Neurology.

[CR130] Wechsler D (2008). Wechsler adult intelligence scale–Fourth Edition (WAIS–IV).

[CR131] Wedekind C, Penn D (2000). MHC genes, body odours, and odour preferences. Nephrology Dialysis Transplantation.

[CR132] Weeks SJ, Hobson RP (1987). The salience of facial expression for autistic children. Journal of Child Psychology and Psychiatry.

[CR133] Wicker B, Monfardini E, Royet JP (2016). Olfactory processing in adults with autism spectrum disorders. Molecular Autism.

[CR134] Williams White S, Keonig K, Scahill L (2007). Social skills development in children with autism spectrum disorders: A review of the intervention research. Journal of Autism and Developmental Disorders.

[CR135] Wysocki CJ, Preti G (2004). Facts, fallacies, fears, and frustrations with human pheromones. The Anatomical Record. Part A, Discoveries in Molecular, Cellular, and Evolutionary Biology.

[CR136] Zamzow RM, Christ SE, Saklayen SS, Moffitt AJ, Bodner KE, Higgins KF (2014). Effect of propranolol on facial scanning in autism spectrum disorder: A preliminary investigation. Journal of Clinical and Experimental Neuropsychology.

[CR137] Zwaigenbaum L, Bauman ML, Choueiri R, Fein D, Kasari C, Pierce K (2015). Early Identification and Interventions for autism spectrum disorder: Executive summary. Pediatrics.

[CR138] Zwaigenbaum L, Bryson S, Garon N (2013). Early identification of autism spectrum disorders. Behavioural Brain Research.

