# Supplementary material for: Exploring Social Biomarkers in High-Functioning Adults with Autism and Asperger’s Versus Healthy Controls: A Cross-Sectional Analysis
Source: J Autism Dev Disord. 2020 Apr 11;50(12):4412–30. doi: 10.1007/s10803-020-04493-5 (PMC7677266; doi:10.1007/s10803-020-04493-5)
Supplement: Supplementary file 1 — Supplementary file1 (DOCX 27 kb) [file 10803_2020_4493_MOESM1_ESM.docx]

**Exploring social biomarkers in high-functioning adults with autism and Asperger’s versus healthy controls: a cross-sectional analysis**

**Supplementary material**

Journal of Autism and Developmental Disorders

Marta del Valle Rubido^1^, Eric Hollander^2^, James T. McCracken^3^, Frederick Shic^4,5^, Jana Noeldeke^1^, Lauren Boak^6^, Omar Khwaja^1^, Shamil Sadikhov^1^, Paulo Fontoura^6^, Daniel Umbricht^1^

1. Roche Pharmaceutical Research and Early Development NORD, Roche Innovation Center, Basel, Switzerland

2. Psychiatry and Behavioral Sciences, Albert Einstein College of Medicine and Montefiore Medical Center, Bronx, New York, USA

3. Psychiatry and Behavioral Sciences, David Geffen School of Medicine at UCLA, Los Angeles, USA

4. Center for Child Health, Behavior and Development, Seattle Children's Research Institute, Seattle, WA, USA

5. Department of Pediatrics, University of Washington, Seattle, WA, USA

6. Roche Product Development Neuroscience, Basel, Switzerland

Corresponding author: Marta del Valle Rubido; [marta.del_valle_rubido@roche.com](mailto:marta.del_valle_rubido@roche.com)

***ESM Table 1.*** Inclusion and exclusion criteria

| **Interventional study** | | | **Observational study** | | |
| --- | --- | --- | --- | --- | --- |
| **ASD** | | | **Healthy controls** | | |
| **Inclusion criteria** | **Exclusion criteria** | **Inclusion criteria** | **Exclusion criteria** | **Inclusion criteria** | **Exclusion criteria** |
| Individuals with a diagnosis of Autistic Disorder or Asperger’s Syndrome as defined by DSM-IV-TR, confirmed by the site’s clinical team and supported by the Autism Diagnostic Observation Schedule (ADOS) | Unwilling to use an effective form of contraception as deemed appropriate to individual patient by the investigator (for example abstinence or condoms) for the duration of the study and for at least 7 days after the last dose | Individuals with a diagnosis of Autistic Disorder or Asperger’s Syndrome as defined by DSM-IV, confirmed by the site’s clinical team and supported by the Autism Diagnostic Observation Schedule (ADOS) |  | – | History or family history (first degree relative)of or current psychiatric, neurological or pervasive developmental disorder |
| Male adults aged 18 to 45 inclusive | Positive test for drugs of abuse | Male Adults (age 18 to 45) | Positive test for drugs of abuse or alcohol | Healthy male adults (age 18 to 45) | Positive test for drugs of abuse or alcohol |
| IQ>70 | Alcohol and/or substance abuse/dependence during the last 12 months | IQ>70 (Wechsler Abbreviated Scale of Intelligence [WASI]) | Alcohol and/or substance abuse/dependence during the last 12 months | IQ>70 | Alcohol and/or substance abuse/dependence during the last 12 months |
| Body mass index between 18 to 35 kg/m^2^ inclusive | A current (at screening) significant risk of suicidal behavior as judged by the Investigator following a thorough clinical evaluation and supported by information collected on the Columbia-Suicide Severity Rating Scale (C-SSRS) | Body mass index between 18 and 35 kg/m^2^ (inclusive) |  | BMI between 18 to 30 kg/m^2^ inclusive |  |
| Behavior Checklist (ABC)-Irritability subscale score ≤13 | Positive result on hepatitis B (HBV), hepatitis C (HCV), or human immunodeficiency virus (HIV) 1 and 2 |  |  |  |  |
| Language, hearing and vision compatible with the study measurements as judged by the investigator | Confirmed systolic blood pressure (SBP) greater than 140 or less than 90 mm Hg, and diastolic blood pressure (DBP) greater than 90 or less than 50 mm Hg. | Language, hearing and vision compatible with the study measurements as judged by the investigator | Confirmed (e.g. 2 consecutive measurements) systolic blood pressure (SBP) greater than 140 or less than 90 mm Hg, and diastolic blood pressure (DBP) greater than 90 or less than 50 mm Hg. | Language, hearing and vision compatible with the study measurements as judged by the investigator | Confirmed (e.g., two consecutive measurements) SBP greater than 140 or less than 90 mmHg, and DBP greater than 90 or less than 50 mmHg |
| Existing medication regimens should be stable for 4 weeks, with the intent to remain stable throughout the study ( | Confirmed resting pulse rate (PR) greater than 100 or less than 45 beats per minute (bpm). | Existing medication regimens should be stable for an appropriate duration as deemed by the investigator prior to Day 1. | Confirmed resting pulse rate (PR) greater than 100 or less than 40 beats per minute (bpm) | – | Resting PR greater than 100 or less than 40 bpm |
| Able to participate and willing to give written informed consent and to comply with the study restrictions | Confirmed clinically significant abnormality on 12-lead electrocardiogram (ECG), including a QT of ≥500 milliseconds. | Able to participate and willing to give written informed consent and to comply with the study restrictions |  | Able to participate and willing to give written informed consent and to comply with the study restrictions |  |
| The availability of a reliable caregiver, able and willing to provide information regarding the individual’s behavior and symptoms | Clinically significant abnormalities in laboratory test results (including hepatic and renal panels, complete blood count, chemistry and coagulation panel and urinalysis). | The presence of a reliable caregiver, able and willing to provide information regarding the individual’s behavior and symptoms |  | – | Clinically significant abnormalities in laboratory test results |
|  | Active stomach ulcer disease, history of coagulopathies or bleeding disorders. |  |  |  |  |
|  | Active inflammatory pulmonary disease. |  |  |  |  |
|  | History of epilepsy/seizure disorder (except simple febrile seizures) |  | History of epilepsy/seizure disorder (except simple febrile seizures) |  |  |
|  | Significant disruptive, aggressive or self-injurious, or sexually inappropriate behavior during the last 3 months that in the opinion of the investigator might interfere with the conduct of the study |  | Significant disruptive, aggressive or self-injurious, or sexually inappropriate behavior during the last 3 months that in the opinion of the investigator might interfere with the conduct of the study |  |  |
|  | Any significant uncontrolled or any unstable medical condition other than Autistic Disorder (e.g. diabetes) that might interfere with the conduct of the study, confound interpretation of the study results, or endanger the subject’s well-being. |  | Concomitant disease or condition that could interfere with, or treatment of which might interfere with, the conduct of the study, or that would, in the opinion of the investigator, pose an unacceptable risk to the subject in this study |  | Concomitant disease or condition that could interfere with, or treatment of which might interfere with, the conduct of the study, or that would, in the opinion of the investigator, pose an unacceptable risk to the subject in this study |
|  | Initiation of new or major change in psychosocial intervention within 4 weeks prior to randomization. Minor changes in ongoing treatment (e.g., missed therapy sessions due to holiday/vacation; planned break in therapy due to school holidays; changes in college/school programs) are not considered significant. |  |  |  |  |
|  | Currently receiving treatment with prohibited medications and not willing to cease treatment for the minimum time period before randomization. |  |  | – |  |
|  | Treatment with any investigational agent within 90 days prior to screening. |  |  |  |  |
|  | Donation of blood over 500 mL within three months prior to screening |  | Donation of blood over 500 mL within three months prior to screening | – | Donation of blood over 500 mL within three months prior to screening |
|  | History of hypersensitivity or allergic reactions |  | History of hypersensitivity or allergic reactions. | – | History of hypersensitivity or allergic reactions |
|  | Lack of peripheral venous access |  | Lack of peripheral venous access | – | Lack of peripheral venous access |
