# Supplementary material for: Exploring Social Biomarkers in High-Functioning Adults with Autism and Asperger’s Versus Healthy Controls: A Cross-Sectional Analysis
Source: J Autism Dev Disord. 2020 Apr 11;50(12):4412–30. doi: 10.1007/s10803-020-04493-5 (PMC7677266; doi:10.1007/s10803-020-04493-5)
Supplement: Supplementary file 2 — Supplementary file2 (PDF 401 kb) [file 10803_2020_4493_MOESM2_ESM.pdf]

Protocol: BP28421 & BP27801
Summary of Statistical Analysis: Treatment comparison as estimated from ANCOVA
Assessment details=Autism-Spectrum Quotient
Covariate VIQ

	LSMean ASD Participants	*LSMean Healthy Controls*	*Estimate ASD Participants - Healthy Controls*	*90% CI*		*p-value*
				Lower	*Upper*	
Total score	29.002	13.735	15.268	11.076	19.460	<0.001

Protocol: BP28421 & BP27801
Summary of Statistical Analysis: Treatment comparison as estimated from ANCOVA
Assessment details=Affective Speech Recognition (ASR)
Covariate VIQ

	LSMean ASD Participants	*LSMean Healthy Controls*	*Estimate ASD Participants - Healthy Controls*	*90% CI*		*p-value*
				Lower	*Upper*	
% (Angry)	55.429	56.381	-0.952	-13.521	11.617	0.900
% (Disgust)	61.590	70.256	-8.666	-20.529	3.197	0.226
% (Fearful)	56.285	47.857	8.428	-3.683	20.539	0.249
% (Happy)	51.607	62.744	-11.137	-22.067	-0.207	0.094
% (Lust)	56.067	57.326	-1.259	-13.044	10.526	0.859
% (Negative emotions)	53.862	56.077	-2.215	-11.195	6.765	0.681
% (Neutral)	67.790	76.060	-8.271	-20.375	3.834	0.258
% (Positive emotions)	54.085	56.081	-1.996	-10.247	6.254	0.687
% (Sad)	58.130	59.643	-1.513	-15.573	12.546	0.857
% (Surprise)	63.759	54.479	9.280	-0.993	19.554	0.136
% Correct answers	55.263	58.063	-2.801	-9.707	4.106	0.500
Tot n. (Angry)	3.326	3.383	-0.057	-0.811	0.697	0.900
Tot n. (Disgust)	3.695	4.215	-0.520	-1.232	0.192	0.226
Tot n. (Fearful)	3.377	2.871	0.506	-0.221	1.232	0.249
Tot n. (Happy)	3.096	3.765	-0.668	-1.324	-0.012	0.094
Tot n. (Lust)	3.364	3.440	-0.076	-0.783	0.632	0.859
Tot n. (Neg.emotions)	12.927	13.458	-0.532	-2.687	1.624	0.681
Tot n. (Neutral)	4.067	4.564	-0.496	-1.222	0.230	0.258
Tot n. (Positive emotions)	9.735	10.095	-0.359	-1.844	1.126	0.687
Tot n. (Sad)	3.488	3.579	-0.091	-0.934	0.753	0.857
Tot n. (Surprise)	3.826	3.269	0.557	-0.060	1.173	0.136
Tot n. correct answers	26.526	27.870	-1.344	-4.660	1.971	0.500

Protocol: BP28421 & BP27801
Summary of Statistical Analysis: Treatment comparison as estimated from ANCOVA
Assessment details=Eye Tracking - Activity Monitoring
Covariate VIQ

	LSMean ASD Participants	*LSMean Healthy Controls*	*Estimate ASD Participants - Healthy Controls*	*90% CI*		*p-value*
				Lower	*Upper*	
Activity (ratio)	0.394	0.317	0.077	0.012	0.142	0.054
Background (ratio)	0.110	0.114	-0.004	-0.028	0.020	0.796
Background wo distractors (ratio)	0.244	0.234	0.010	-0.038	0.058	0.732
Body (ratio)	0.131	0.117	0.014	-0.020	0.049	0.489
Distractors (ratio)	0.134	0.120	0.014	-0.026	0.053	0.563
Head (ratio)	0.231	0.332	-0.101	-0.153	-0.050	0.002
Person (ratio)	0.362	0.449	-0.087	-0.141	-0.033	0.010

Protocol: BP28421 & BP27801
Summary of Statistical Analysis: Treatment comparison as estimated from ANCOVA
Assessment details=Eye Tracking – Biodetection
Covariate VIQ

	LSMean ASD Participants	*LSMean Healthy Controls*	*Estimate ASD Participants - Healthy Controls*	*90% CI*		*p-value*
				Lower	*Upper*	
D-prime (masked cond.)	1.256	1.689	-0.432	-1.023	0.159	0.226

Protocol: BP28421 & BP27801
Summary of Statistical Analysis: Treatment comparison as estimated from ANCOVA
Assessment details=Eye Tracking – Biomotion
Covariate VIQ

	LSMean ASD Participants	*LSMean Healthy Controls*	*Estimate ASD Participants - Healthy Controls*	*90% CI*		*p-value*
				Lower	*Upper*	
Latency (ms)	301.057	262.949	38.107	-9.811	86.026	0.189
Looking pref (ratio)	0.545	0.620	-0.076	-0.130	-0.021	0.023
Orienting pref (ratio)	0.484	0.492	-0.008	-0.041	0.025	0.672

Protocol: BP28421 & BP27801
Summary of Statistical Analysis: Treatment comparison as estimated from ANCOVA
Assessment details=Eye Tracking – WAVW
Covariate VIQ

	LSMean ASD Participants	*LSMean Healthy Controls*	*Estimate ASD Participants - Healthy Controls*	*90% CI*		*p-value*
				Lower	*Upper*	
Background (ratio)	0.114	0.100	0.014	-0.010	0.038	0.323
Body (ratio)	0.098	0.079	0.019	-0.007	0.045	0.222
Eyes (ratio)	0.417	0.447	-0.031	-0.117	0.055	0.551
Head (ratio)	0.772	0.813	-0.041	-0.087	0.005	0.145
Mouth (ratio)	0.262	0.292	-0.030	-0.098	0.039	0.469

Protocol: BP28421 & BP27801
Summary of Statistical Analysis: Treatment comparison as estimated from ANCOVA
Assessment details=Eye Tracking - Gaze Discrimination
Covariate VIQ

	LSMean ASD Participants	*LSMean Healthy Controls*	*Estimate ASD Participants - Healthy Controls*	*90% CI*		*p-value*
				Lower	*Upper*	
Eyes (ratio)	0.411	0.415	-0.005	-0.103	0.093	0.934
Inside face (ratio)	0.937	0.954	-0.017	-0.051	0.016	0.391
Mouth (ratio)	0.075	0.070	0.005	-0.032	0.041	0.833
Nose (ratio)	0.297	0.331	-0.034	-0.100	0.032	0.389

Protocol: BP28421 & BP27801
Summary of Statistical Analysis: Treatment comparison as estimated from ANCOVA
Assessment details=Eye Tracking - Gender Discrimination
Covariate VIQ

	LSMean ASD Participants	*LSMean Healthy Controls*	*Estimate ASD Participants - Healthy Controls*	*90% CI*		*p-value*
				Lower	*Upper*	
Eyes (ratio)	0.236	0.191	0.045	-0.031	0.120	0.324
Inside face (ratio)	0.938	0.941	-0.003	-0.044	0.038	0.909
Mouth (ratio)	0.122	0.112	0.011	-0.033	0.055	0.682
Nose (ratio)	0.411	0.465	-0.054	-0.125	0.017	0.211

Protocol: BP28421 & BP27801
Summary of Statistical Analysis: Treatment comparison as estimated from ANCOVA
Assessment details=Eye Tracking – Pupillometry
Covariate VIQ

	LSMean ASD Participants	*LSMean Healthy Controls*	*Estimate ASD Participants - Healthy Controls*	*90% CI*		*p-value*
				Lower	*Upper*	
from Activity Monitoring (mm)	3.710	3.343	0.367	0.087	0.647	0.033
from Biodetection (mm)	4.514	4.126	0.388	-0.021	0.797	0.118
from Biomotion (mm)	4.270	3.968	0.301	-0.006	0.608	0.106
from Gaze discr. (mm)	3.666	3.313	0.354	0.067	0.640	0.044
from Gender discr. (mm)	3.667	3.312	0.355	0.069	0.641	0.043
from Human activity (mm)	3.711	3.458	0.253	-0.029	0.535	0.139
from WAVW (mm)	4.045	3.674	0.371	0.071	0.672	0.044

Protocol: BP28421 & BP27801
Summary of Statistical Analysis: Treatment comparison as estimated from ANCOVA
Assessment details=Eye Tracking - Human activity
Covariate VIQ

	LSMean ASD Participants	*LSMean Healthy Controls*	*Estimate ASD Participants - Healthy Controls*	*90% CI*		*p-value*
				Lower	*Upper*	
Preference (ratio)	0.563	0.649	-0.086	-0.199	0.028	0.211

Protocol: BP28421 & BP27801
Summary of Statistical Analysis: Treatment comparison as estimated from ANCOVA
Assessment details=Eye Tracking – Total
Covariate VIQ

	LSMean ASD Participants	*LSMean Healthy Controls*	*Estimate ASD Participants - Healthy Controls*	*90% CI*		*p-value*
				Lower	*Upper*	
Composite score	-0.167	0.241	-0.407	-0.870	0.056	0.147

Protocol: BP28421 & BP27801
Summary of Statistical Analysis: Treatment comparison as estimated from ANCOVA
Assessment details=Read the Mind in the Eyes Test (RMET)
Covariate VIQ

	LSMean ASD Participants	*LSMean Healthy Controls*	*Estimate ASD Participants - Healthy Controls*	*90% CI*		*p-value*
				Lower	*Upper*	
% correct answers	66.384	76.014	-9.630	-19.061	-0.199	0.093
Tot n. correct answers	23.898	27.365	-3.467	-6.862	-0.072	0.093

Protocol: BP28421 & BP27801
Summary of Statistical Analysis: Treatment comparison as estimated from ANCOVA
Assessment details=Smell Test
Covariate VIQ

	LSMean ASD Participants	*LSMean Healthy Controls*	*Estimate ASD Participants - Healthy Controls*	*90% CI*		*p-value*
				Lower	*Upper*	
% correct answers	85.163	89.858	-4.694	-11.910	2.521	0.279
Tot n. correct answers	10.219	10.781	-0.562	-1.428	0.303	0.280

Protocol: BP28421 & BP27801
Summary of Statistical Analysis: Treatment comparison as estimated from ANCOVA
Assessment details=STAI scale
Covariate VIQ

	LSMean ASD Participants	*LSMean Healthy Controls*	*Estimate ASD Participants - Healthy Controls*	*90% CI*		*p-value*
				Lower	*Upper*	
Total score	38.916	30.641	8.276	1.516	15.035	0.045
